# Parental and offspring contribution of genetic markers of adult blood pressure in early life: The FAMILY study

**DOI:** 10.1371/journal.pone.0186218

**Published:** 2017-10-18

**Authors:** Sébastien Robiou-du-Pont, Sonia S. Anand, Katherine M. Morrison, Sarah D. McDonald, Stephanie A. Atkinson, Koon K. Teo, David Meyre

**Affiliations:** 1 Department of Health Research Methods, Evidence, and Impact, McMaster University, Hamilton, Ontario, Canada; 2 Department of Medicine, McMaster University, Hamilton, Ontario, Canada; 3 Department of Pediatrics, Hamilton Health Sciences and McMaster University, Hamilton, Ontario, Canada; 4 Department of Obstetrics and Gynecology, McMaster University, Hamilton, Ontario, Canada; 5 Department of Pathology and Molecular Medicine, McMaster University, Hamilton, Ontario, Canada; Universite de Montreal, CANADA

## Abstract

Previous genome wide association studies (GWAS) identified associations of multiple common variants with diastolic and systolic blood pressure traits in adults. However, the contribution of these loci to variations of blood pressure in early life is unclear. We assessed the child and parental contributions of 33 GWAS single-nucleotide polymorphisms (SNPs) for blood pressure in 1,525 participants (515 children, 406 mothers and 237 fathers) of the Family Atherosclerosis Monitoring In early life (FAMILY) study followed-up for 5 years. Two genotype scores for systolic (29 SNPs) and diastolic (24 SNPs) blood pressure were built. Linear mixed-effect regressions showed significant association between rs1378942 in *CSK* and systolic blood pressure (β = 0.98±0.46, *P* = 3.4×10^−2^). The child genotype scores for diastolic and systolic blood pressure were not associated in children. Nominally significant parental genetic effects were found between the SNPs rs11191548 (*CYP17A1*) (paternal, β = 2.78±1.49, *P* = 6.1×10^−2^ for SBP and β = 3.60±1.24, *P* = 3.7×10^−3^ for DBP), rs17367504 (*MTHFR*) (paternal, β = 2.42±0.93, *P* = 9.3×10^−3^ for SBP and β = 1.89±0.80, *P* = 1.8×10^−2^ for DBP and maternal, β = -1.32±0.60, *P* = 2.9×10^−2^ and β = -1.97±0.77, *P* = 1.0×10^−2^, for SBP and DBP respectively) and child blood pressure. Our study supports the view that adult GWAS loci have a limited impact on blood pressure during the five first years of life. The parental genetic effects observed on blood pressure in children may suggest epigenetic mechanisms in the transmission of the risk of hypertension. Further replication is needed to confirm our results.

## Introduction

In 2008, 978 million adults, or 28% of the global adult population had hypertension (HTN) and the burden of HTN may reach 1.5 billion by 2025 [1,2]. HTN is associated with an increased risk for cardiovascular disease and contributes as such to 7.6 million (13.5%) deaths each year worldwide [1]. Modifiable risk factors for HTN include excessive dietary sodium, physical inactivity, excessive alcohol intake, psychosocial stress and obesity [3]. Non modifiable risk factors include sex, age, but also ethnicity and family history of HTN, suggesting a contribution of genetic determinants in HTN etiology [4]. Twin and family studies have reported heritability estimates of 30–50% for blood pressure (BP) and hypertension [5]. Twelve genes have been associated with Mendelian syndromes causing HTN [5]. Genome-wide association studies (GWAS) have identified 54 common genetic variants associated with systolic blood pressure (SBP) and diastolic blood pressure (DBP) [5,6]. These GWAS signals point toward the role of vasodilatory hormones, ionic regulation by solute channels and vascular smooth muscle growth and signaling in the pathogenesis of HTN [7]. It is noteworthy that most of the GWAS studies for BP have been performed in adults of European ancestry, and only one GWAS for BP has been reported in children and adolescents [5,8,9]. To date, four studies assessed the contribution of SNPs identified in adult GWAS in children and adolescents of European ancestry [9–12]. Oikonen *et al*. built two genotype scores by using 5 SBP and 8 DBP-associated SNPs and did not find any evidence of association with SBP and DBP from the age of 3 to 18 years (sample size comprised between 340 and 1100) [10]. More recently, Howe *et al*. studied a unique genotype score based on 29 adult BP SNPs in 8472 children from Australia and United Kingdom and evidenced a nominal association only with SBP at the ages of 6 and 17 years [11]. Early 2016, an international consortium found two novel loci associated with SBP at pre-puberty (4–7 years) and puberty (8–12 years-) [9]. The authors also highlighted an age specific association of the two SNPs.

Parental history of high BP has been associated with higher SBP and DBP in offspring in the literature; some but not all studies reporting sex-specific parental effects [13,14]. Family heritability studies for SBP and DBP support the view that the phenotypic resemblance observed between parents and offspring may be explained in part by genetic determinants [15,16]. However, the parental contribution of genetic markers of adult BP in offspring has never been investigated.

This prompted us to investigate the parental and child contributions of 33 GWAS associated-SNPs for BP in 1,525 participants of the Family Atherosclerosis Monitoring In early life (FAMILY) study followed-up from birth to the age of 5 years.

## Methods

### Subjects

The Family Atherosclerosis Monitoring In earLY life (FAMILY) study has been described elsewhere [17]. FAMILY is an ongoing birth cohort study that includes mothers, fathers and children with a planned follow-up of 10 years. Briefly, over the last 7 years, 859 families including 901 babies, 259 siblings, 857 mothers and 530 fathers were enrolled into the FAMILY study. In this study, we excluded offspring from multiple births, siblings of “index” children due to familial relatedness and phenotypic issues (i.e. absence of phenotypic data at birth). Following these exclusion criteria, 630 mothers, 351 fathers and 544 unrelated children had DNAs extracted and were selected for genotyping. After assessing the family structure between the children and their parents, we selected 515 children, 406 mothers and 237 fathers with genotypic and phenotypic (sex, age and BMI) data for the analysis (406 child/mother pairs, 237 child/father pairs, and 219 trios). Phenotypic characteristics of these individuals are available in the Table 1. Sample sizes at each time of measurement are available in the S1 Fig. The data coordination site of the FAMILY study is the Population Health Research Institute (Hamilton, ON, Canada). Informed consent was obtained from all the adult participants, and the parents provided consent for their children. All procedures were performed in accordance with relevant guidelines and regulations. The study was approved by the Research Ethics Boards at the participating hospitals (Hamilton Health Sciences, St Joseph’s Hospital—Hamilton, Joseph Brant Memorial Hospital, Burlington, ON, Canada).

**Table 1:** Phenotypic characteristics of the studied population.

	Visit	Father	Mother	Child	
				Sample size	
N		351	630		544
% male		100%	0%		47.67%
Age (years)	Birth visit	33.92 ± 5.65	32.31 ± 4.67	515	0.007 ± 0.013
	1	NA	33.79 ± 4.61	487	1.08 ± 0.13
	2	NA	NA	470	2.07 ± 0.12
	3	NA	NA	441	3.08 ± 0.16
	5	40.03 ± 5.80	38.36 ± 4.55	383	5.12 ± 0.19
BMI (kg/m^2^)	Initial visit	28.39 ± 4.76	26.73 ± 6.27	NA	NA
	Birth visit	NA	NA	515	13.99 ± 1.39
	1	NA	26.99 ± 6.68	487	17.54 ± 1.42
	2	NA	NA	470	16.39 ± 1.26
	3	NA	27.646 ± 6.30	441	16.17 ± 1.20
	5	28.45 ± 4.83	27.40 ± 6.640	383	15.87 ± 1.59
SBP (mmHg)	Initial visit	124.5 ± 10.4	113.6 ± 10.6	NA	NA
	Birth visit	NA	NA	181	68.9 ± 10.7
	1	NA	113.5 ± 10.3	318	96.6 ± 11.5
	2	NA	NA	391	96.6 ± 10.6
	3	NA	NA	421	95.8 ± 8.5
	5	124.9 ± 11.3	113.9 ± 12.8	379	99.0 ± 8.3
DBP (mmHg)	Initial visit	79.0 ± 9.6	70.9 ± 8.4	NA	NA
	Birth visit	NA	NA	454	39.2 ± 8.6
	1	NA	73.5 ± 9.1	316	59.6 ± 7.6
	2	NA	NA	391	60.5 ± 7.0
	3	NA	NA	421	60.2± 5.9
	5	78.0 ± 9.7	73.5 ± 10.3	379	60.2 ± 5.6

BMI, body mass index. DBP, diastolic blood pressure. SBP, systolic blood pressure. NA, data not available.

### Phenotyping

Offspring’s phenotypic measurements have been performed at birth, 1 year, 2 years, 3 years and 5 years of age (Table 1). Systolic and diastolic blood pressures were measured with a Dinamap Pro100 V2 (GE Medical Systems, Tampa, Florida, USA), which utilizes an oscillometric method, and repeated 3 times at 2 minutes intervals. At birth the measures were performed while the child was sleeping or lying quietly. For all other time measurement (1, 2, 3 and 5 years), the measures were performed while the child was sitting quietly and after resting for at least 5 minutes. The child’s height was recorded from birth to 2 years using an O'Leary pediatric length board (Ellard Inc) then using Harpenden stadiometer with a precision of 0.1 cm. The weight was measured to the nearest 200g in light clothes using an electronic top. BMI was calculated using the following formula: weight (kg)/ height^2^ (m^2^).

### Genotyping

Genomic DNAs were extracted from buffy coats for all the participants. Buffy coats for mothers and fathers were extracted from blood samples collected at the initial visit at the 24–37 weeks of gestation. For the child, the buffy coat comes from cord blood at the delivery. The genotyping was performed using the Illumina Cardio-Metabochip (San Diego, CA, USA). This array has been designed by seven consortia on cardiac, metabolic and anthropometric traits. A selection of 196,725 SNPs for 23 different traits was made. The design and SNP selection of the array have been detailed elsewhere [18]. We selected SNPs that reached genome-wide significance level of association (*P*<5×10^−8^) for SBP and/or DBP in at least one population of European ancestry and were available in the Cardio-Metabochip array (lead SNP or proxy). All the SBP and DBP-associated SNPs were extracted from two databases (HuGE Navigator and NHGRI GWAS Catalog). For SNPs that were not available in the Cardio-Metabochip, we searched for proxy SNPs using the Broad Institute website tool SNAP (SNP Annotation and Proxy Search). For those highlighted as missing in the Cardio-Metabochip, we checked their availability using their chromosomal position in the Illumina product file. We used the following criteria to select proxy SNPs: 1) SNPs included in the Cardio-Metabochip 2) r^2^ >0.95 in European population data issued from the 1000 Genomes Project, 3) selection of a coding non-synonymous SNP if available in the list of proxy, otherwise selection of the SNP located closest to the GWAS lead SNP. To avoid any overlap in the final SNP selection, linkage disequilibrium between all the SNPs was double-checked using SNAP in European population data of the 1000 Genomes Project. We discarded 13 SNPs that displayed r^2^ > 0.2 with another SNP in the list. Thirty-three SBP and DBP-associated polymorphisms remains for further study (S1 Table). Standard procedures have been used to assess the quality of the genotyping: all 33 SNPs displayed call rates > 99% and are consistent with the Hardy Weinberg Equilibrium (S2 and S3 Tables). As an additional quality control procedure we analyzed the Mendelian transmission patterns of the 33 SNPs. We found recurrent Mendelian inconsistencies in five pedigrees. After excluding the five non-biological fathers from the analysis, only one Mendelian distortion was observed in the whole sample for the 33 SNPs, which therefore successfully passed the quality control test. Data from the five non-biological fathers were excluded from further analyses. We then searched for discrepancies between the reported sex and the one determined using the genetic information. We found 9 discrepancies by using the heterozygosity rate calculated by PLINK. The cryptic relatedness between the children was also verified and we removed six individuals due to evidence of relatedness (second degree relatives). We double-checked the self-reported ethnicity of our individuals using EIGENSTRAT. The 1525 participants of the FAMILY study were predominantly white Caucasians (92.8% Mothers; 89.3% Fathers; 91.1% Offspring). Other participants were South Asians, East Asians, Latino Americans, Africans and Native North Americans.

### Statistical analyses

S1 and S2 Files provide datasets for SBP and DBP analyses, respectively. We coded genotypes as 0, 1 and 2, depending on the number of copies of the SBP or DBP increasing alleles. Two genotype scores were calculated by summing the alleles of 24 and 29 SNPs for SBP and DBP, respectively. The genotype scores were used as an ordinal value in the models. Considering the possibility that genetic effects for BP GWAS SNPs may diverge in adult and children populations, we used an unweighted genotype score to prevent any analytical bias. Unweighted and weighted genotype scores for complex traits usually have a comparable performance [19,20]. This is especially true if the differences in genetic effects of SNPs are minor and if the sample size is not very large, two conditions that apply to our study [19,20]. Individuals with more than two missing values were discarded from the calculation of the genotype score and the remaining missing values were imputed using the method of the mean. This imputation was performed for each SNP individually using the arithmetic average of the coded genotypes observed for all the successfully genotyped individuals.

We did not perform family-based association tests in this study for two reasons. First, larger sample sizes are needed for family-based than regression association tests to achieve comparable statistical power [21]. The low participation rate of fathers in the FAMILY study (515 children, 406 mothers and 237 fathers) adds to the loss of statistical power. Unfortunately, this is a common pitfall of family-based designs where mothers often bring children to clinic visits and thus are included more easily than fathers [22]. Second, the software used in family-based association tests only perform cross-sectional analyses. Longitudinal analyses have been shown to achieve more power than cross-sectional association tests [23,24]. Associations between SNP/genotype scores and BP measurements were assessed using linear mixed-effect regression model to account for the longitudinal nature of the data (5 SBP and DBP measurements). We used the intercept and the age at measurement as random effects and sex, BMI and the principal components as fixed effects. To assess paternal and/or maternal effects on offspring’s SBP and DBP, a linear mixed-effect regression was performed using the parental genetic information (SNPs or genotype score) as predictor and sex, age, BMI, principal components and SNPs/genotype score of the offspring as covariates. The 10 first principal components were computed using all the SNPs passing the quality control filter in the Metabochip and they were defined using EIGENSTRAT [25]. Principal components were added as covariates in all regression models to account for population structure. We handled SBP and DBP missing data at different ages through a missing at random approach in the linear mixed-effect regression model and did not to impute SBP and DBP missing data in our study. This decision was based on three arguments: 1) the percentage of SBP and DBP missing data at each measurement is heterogeneous in FAMILY (S1 Fig); 2) SBP and DBP values vary significantly in early life and a large inter-individual variability is observed at each measurement (S2 Fig); 3) linear mixed-effect regression models handle well the presence of missing data [24]. All the regression analyses were performed using the free software R 3.0.1 with the package lme4 [26].

Hardy-Weinberg equilibrium was tested using a Chi-square test in combination with permutations and bootstrapping. Mendelian incompatibilities were checked using PLINK [27]. Two-tailed P-values are presented in this manuscript. Bonferroni corrected P-values are routinely applied to exploratory genetic association studies. However, they are overly conservative given the high prior likelihood of association in post-GWAS experiments. *P* < 0.05 was therefore considered significant for post-GWAS associations between offspring SNPs/GSs and BP traits in children. We did not apply a Bonferroni correction for the comparison of genetic effects of SNPs at different ages in children, and between children and adults, as they represent *post-hoc* analyses for BP-associated SNPs. In contrast, we applied Bonferroni corrections for the exploratory associations of 1) paternal SNPs/GSs and 2) maternal SNPs/GSs with BP traits in children, as no evidence of parent-of-origin effects on BP traits has been reported in literature before. *P* <2.0×10^−3^ (0.05/25) and *P* <1.7×10^−3^ (0.05/30) was considered as significant for SBP and DBP respectively. We previously applied a similar approach for the study of obesity traits in FAMILY [28]. We compared our significant mixed model results with those obtained on an adult cohort from the International Consortium for Blood Pressure using a Z-test [29,30]. We also performed Z-tests on the child beta values across time to assess potential age-dependent genetic effects.

## Results

### Evolution of the phenotypes across the follow up

SBP and DBP increased during the first year of life to reach a plateau until the age of 5 years (S2 Fig).

### Associations of offspring SNPs and genotype scores with blood pressure in children

Linear mixed-effect regressions on the longitudinal series of data were used to assess the effect of children’s SNPs on SBP and DBP from birth to 5 year (S4 and S5 Tables). The rs11191548 SNP near *CYP17A1* showed directionally consistent association with DBP (β = 1.71±0.61, *P* = 4.6×10^−3^) (Table 2 and S5 Table). The rs1378942 in *CSK* showed directionally consistent association with SBP (β = 0.98±0.46, *P* = 3.4×10^−2^). Directionally inconsistent association was found for the rs12946454 in *PLCD3* and SBP (β = -1.07±0.50, *P* = 3.3×10^−2^) (Table 2 and S4 Table). To assess the combined effect of the SBP and DBP SNPs, we tested the association of the children’s genotype score using a linear mixed-effect regression model on the longitudinal series of data (S4 and S5 Tables). Neither the SBP nor DBP genotype scores showed associations with SBP or DBP.

**Table 2:** Summary of the significant results using mixed-effect regressions.

Effect	Trait	GENE	SNP	R.A.	BETA	SE	*P*
Children	DBP	*CYP17A1*	rs11191548	A	1.713	0.605	4.61×10^−3^
	SBP	*CSK*	rs1378942	C	0.979	0.462	3.42×10^−2^
	SBP	*PLCD3*	rs12946454	T	-1.067	0.501	3.34×10^−2^
Maternal	DBP	*MTHFR*	rs17367504	A	-1.320	0.600	2.78×10^−2^
	DBP	*ULK4*	rs1717017	C	1.384	0.562	1.39×10^−2^
	DBP	*EBF1*	rs12187017	G	-1.013	0.466	2.98×10^−2^
	SBP	*MTHFR*	rs17367504	A	-1.974	0.770	1.04×10^−2^
	SBP	*PLEKHA7*	rs381815	A	1.314	0.637	3.90×10^−2^
Paternal	DBP	*MTHFR*	rs17367504	A	1.888	0.796	1.77×10^−2^
	DBP	*CYP17A1*	rs11191548	A	3.602	1.241	3.70×10^−3^
	SBP	*MTHFR*	rs17367504	A	2.418	0.930	9.28×10^−3^

SNP, Single Nucleotide Polymorphism. SBP, Systolic Blood Pressure. DBP, Diastolic Blood Pressure. R.A., Risk Allele. In the children section, we tested the association of the children SNPs with children BP. In the others section, we assessed the effect of maternal or paternal SNP on children phenotypes.

### Associations of parental SNPs and genotype scores with blood pressure in children

Linear mixed-effect regressions on the longitudinal series of data were used to assess the effect of parental SNPs on SBP and DBP in offspring (S6 and S7 Tables). The regressions of the offspring’s phenotypes highlighted a directionally consistent nominal evidence of association of the paternal genotype of rs11191548 (*CYP17A1*) for DBP (β = 3.60±1.24, *P* = 3.7×10^−3^) and a trend of association with SBP after adjusting for offspring’s genotypes (β = 2.78±1.49, *P* = 6.1×10^−2^). Further adjustment for the maternal genotype did not significantly modify the nominal association of the paternal genotype of rs11191548 (*CYP17A1*) with SBP and DBP (β = 2.24±1.18, *P* = 5.7×10^−2^ and β = 2.85±1.00, *P* = 4.2×10^−3^, respectively). We did not find any association between the maternal genotype of rs11191548 (*CYP17A1*) and SBP or DBP. The associations of the child genotype rs11191548 (*CYP17A1*) with SBP and DBP did not resist to an adjustment by the paternal genotype (Table 2, S6 and S7 Tables). Both the maternal and paternal genotypes of rs17367504 (*MTHFR*) were nominally associated with SBP and DBP. The nominal associations for SBP and DBP adjusted for the offspring’s genotype were directionally inconsistent when the rs17367504 (*MTHFR*) maternal genotype was assessed (β = -1.32±0.60, *P* = 2.9×10^−2^ and β = -1.97±0.77, *P* = 1.0×10^−2^, respectively). In contrast, these nominal associations were directionally consistent when the paternal genotype was assessed (β = 2.42±0.93, *P* = 9.3×10^−3^ for SBP and β = 1.89±0.80, *P* = 1.8×10^−2^ for DBP). The paternal and maternal nominal associations of rs17367504 (*MTHFR*) with SBP and DBP were removed when the model was adjusted for the reciprocal parental genotype (Table 2, S6 and S7 Tables). The maternal genotype of rs1717017 (*ULK4*) was directionally consistent and nominally associated with DBP after adjusting for the offspring’s genotype (β = 1.38±0.56, *P* = 1.4×10^−2^). The maternal genotype of rs12187017 near *EBF1* was found to be nominally associated in an inconsistent direction with DBP after adjusting for the offspring’s genotype (β = -1.01±0.47, *P* = 3.0×10^−2^). These associations disappeared after adjusting for the corresponding paternal genotype (rs1717017 and rs12187017). We did not find any association between the paternal genotype of rs1717017 (*ULK4*) or rs12187017 (*EBF1*) and DBP (Table 2 and S7 Table). Linear mixed-effect regressions highlighted a nominal and directionally consistent association between the maternal genotype of rs381815 (*PLEKHA7*) and SBP (β = 1.31±0.64, *P* = 3.9×10^−2^) (Table 2 and S6 Table). The maternal and paternal genotype scores were not associated with child SBP or DBP (S6 and S7 Tables). None of the above-mentioned maternal or paternal associations with BP traits in children survived to a Bonferroni correction.

### Age-dependent genetic effects in children

We compared the effect size of the SNPs found to be at least nominally associated with child BP using linear mixed-effect model during the follow-up from birth to 5 year in FAMILY using Z-test calculation between the measurements. Rs1378942 (*CSK*) and rs12946454 (*PLCD3*) SNPs did not show a differential effect from birth to 5 year (*P*_*Z-test*_ = 7.2×10^−2^ and *P*_*Z-test*_ = 0.32, respectively) whereas the rs11191548 SNP (*CYP17A1*) showed a significant decrease in its effect during the 5 years follow-up (β_0_ = 2.09±0.88 *P*_*0*_ = 2.4×10^−3^, β_5_ = 0.32±0.71, *P*_*5*_ = 0.65, *P*_*Z-test*_ = 1.8×10^−2^) (S8 and S9 Tables).

Significantly larger genetic effects were observed in children from FAMILY in comparison with an adult cohort from the International Consortium for Blood Pressure for the following SNPs: *CYP17A1* rs11191548 (DBP) (β_child_ = 1.71±0.61, β_adults_ = 0.52±0.11, *P*_*Z-test*_ = 2.6×10^−2^) and *PLCD3* rs12946454 (SBP) (β_child_ = -1.07±0.50, β_adults_ = 0.60±0.10, *P*_*Z-test*_ = 5.9×10^−4^). In contrast, the *CSK* rs1378942 SNP (SBP) (β_child_ = 0.98±0.46, β_adults_ = 0.63±0.10, *P*_*Z-test*_ = 0.23) did not show age-dependent genetic effects.

We assessed potential changes of the parental effects of rs11191548 (*CYP17A1*) and rs17367504 (*MTHFR*) SNPs from birth to 5 year. The parental genetic effect of these two SNPs on child’s SBP and DBP did not vary during the follow-up.

## Discussion

In this study, we assessed the associations of 33 GWAS SBP/DBP SNPs in the FAMILY birth cohort. The SNP rs1378942 (*CSK*) showed a significant association with SBP from birth to 5 year in line with previous reports on adults [7,29–31]. *CSK* (*c-Src tyrosine kinase*) is a tyrosine kinase with roles in the mediation of the G protein signals to actin cytoskeletal reorganization [32]. Actin remodeling has a direct impact on the constriction of the arterial endothelium in rats and human newborns, supporting genetic effects in early life [33,34]. In line with our data, a nominal association between rs1378942 (*CSK*) and SBP was recently reported in 8,472 children from Australia and United Kingdom at the age of 6 years [11]. An association of rs1378942 *(CSK*) with SBP was also reported in 1,027 Chinese obese children [35]. The fact that *CSK* rs1378942 SNP shows comparable genetic effects on SBP in both FAMILY and adult populations from the International Consortium for Blood Pressure suggests that this SNP contributes equally to SBP variations over the life course. In contrast, the genotype scores based on 24 and 29 SNPs did not show any association with child SBP and DBP in early life in our study. Similarly, Oikonen *et al*. did not evidence any association between two genotype scores based on 5 SBP and 8 DBP-associated SNPs and SBP or DBP from the age of 3 to 18 years [10]. In contrast, Howe *et al*. found a nominal association between a genotype score based on 29 adult SBP/DBP SNPs and SBP at the ages of 6 and 17 years [11]. The inconsistency of this finding with our data may relate to the unique nature of the composite genotype score developed by Howe and colleagues, thus making direct comparison difficult [11]. The lack of association between the SBP and DBP genotype scores observed by us and others [10] during childhood and adolescence is consistent with the fact that heritability estimates for both traits increase progressively during this age window to reach a plateau at young adulthood [36]. Similarly, the association of SNPs such as *CSK* rs1378942 or the genotype score by us and others with child SBP (but not DBP) is in line with the systematically lower heritability estimates found for DBP in comparison with SBP in adolescents and adults [37,38]. As no longitudinal study to date reported heritability estimates for SBP and DBP in the first years of life, we calculated these values in FAMILY and also found a progressive increase of heritability estimates from birth to 5 years and an overall lower heritability for DBP than SBP (DBP: from 1.1 to 26.4%; SBP: from 0.0% to 31.5%; Robiou-du-Pont *et al*., manuscript in preparation). These results deserve further investigations but show that beyond lifestyle a subset of genetic factors already plays a role in early life.

We are aware of the modest power of our study, as showed by our power calculation simulations (S3 Fig). A suboptimal statistical power inflates both the risk of false negative and false positive associations [21]. This means that, in addition to the association observed between *CSK* rs1378942 and SBP, other SNPs contributing to BP in early life may have been missed in the present study. We also speculate that some of the associations observed in this study with BP (e.g. rs12946454 in *PLCD3*) but displaying an inconsistent direction of effect in contrast with previous literature in adults may represent false positive results. Alternatively, we cannot exclude the possibility of age-dependent genetic effects on BP, as recently reported by Simino *et al*. for SBP and DBP in young *versus* old adults [39]. An inversion of genetic effect in infancy *versus* childhood has also been reported for the *FTO* intron 1 variant in relation with body mass index in eight longitudinal cohorts of European ancestry [40].

This study is the first to assess parental genetic effect of SNPs identified by GWAS on SBP and DBP phenotypes in early life independent of the influence of child genotype. This investigation highlighted a nominal association of the paternal BP increasing *CYP17A1* rs11191548 allele with higher child SBP and DBP using a mixed-effect model. Maternal and paternal alleles of rs17367504 in *MTHFR* display opposite effects on BP in children. While the paternal SBP/DBP increasing allele of rs17367504 (*MTHFR*) shows a directionally consistent nominal association with offspring’s BP from birth to 5 years, the maternal SBP/DBP increasing allele at the same SNP is nominally associated with a decrease in children’s BP. Beyond the associations described at the *MTHFR* and *CYP17A1* loci, other nominally significant parental effects were observed for rs12187017 (*EBF1*), rs1717017 (*ULK4*), rs381815 (*PLEKHA7*) and offspring BP. Even if the biology of these genes does not enable a trivial explanation for these associations, further replication of these nominally significant results in additional studies is warranted to definitively assess the potential epigenetic transmission of hypertension.

Our study has several strengths. First and most importantly, this report is the first to investigate SNPs that affect BP from birth to five years. This is also the first time that parental effects are studied on BP in young children. Furthermore, the longitudinal FAMILY study provided a unique opportunity to investigate the effects of parental SNPs on offspring BP using mixed-effects models. Of note, several genetic associations have been strengthened by plausible biological arguments. Lastly, the Illumina Cardio-Metabochip allowed us to investigate the most exhaustive list of SNPs so far (N = 33).

One limitation of the study is the modest sample size, which restricted our power to detect associations with small effect sizes and/or low risk allele frequencies. The longitudinal nature of our study and the use of linear mixed-effect regressions compensated to a certain extent the suboptimal power. Another limitation is the low number of fathers recruited. This however constitutes a common feature of birth cohorts focusing principally on mothers and offspring.

In conclusion, we highlighted in this study a significant association of the rs1378942 SNP in *CSK* with SBP during the first years of life, but no overall association of the GWAS BP SNPs using SBP/DBP genotype scores. Moreover and for the first time, nominally significant parental genetic effects were found between the SNPs rs11191548 (*CYP17A1*) and rs17367504 (*MTHFR*) and child BP suggesting possible epigenetic mechanisms in the transmission of susceptibility to hypertension. Our results suggest that the genetic predisposition for hypertension have a limited impact on BP during the first years. Furthermore, the observation of paternal and maternal genetic effects may contribute to explain why maternal risk factors do not account for the global phenotypic variance of child BP [41].

## Supporting information

S1 File Dataset for systolic blood pressure analyses. (TXT)

S2 File Dataset for diastolic blood pressure analyses. (TXT)

S1 Table Characteristics of the 33 selected SNPs. MAF, Minor Allele Frequency in the studied population. GWAS, Genome-Wide Association Study. SNP, Single Nucleotide Polymorphism. CHR, Chromosome. SBP, Systolic Blood Pressure. DBP, Diastolic Blood Pressure.
(PDF)

S2 Table Genotype count, call rate and Hardy Weinberg Equilibrium (HEW) test for the FAMILY population. SNP: Single Nucleotide Polymorphism. CHR: Chromosome. CR: Call Rate. HWE: P-value resulting of Hardy Weinberg Equilibrium test.
(PDF)

S3 Table Genotype count and Hardy Weinberg Equilibrium test (HWE) for mothers and fathers. (PDF)

S4 Table Results of regression of offspring genotype for systolic blood pressure. A linear regression was performed of the offspring genotype at each time of measurement (birth, 1, 2, 3 and 5y) with sex and BMI as adjustment. The linear mixed-effect regression model was performed of the offspring genotype adjusted by sex and BMI as fixed effect and by the intercept and age as random effect.
(PDF)

S5 Table Results of regression of offspring genotype for diastolic blood pressure. A linear regression was performed of the offspring genotype at each time of measurement (birth, 1, 2, 3 and 5y) with sex and BMI as adjustment. The linear mixed-effect regression model was performed of the offspring genotype adjusted by sex and BMI as fixed effect and by the intercept and age as random effect.
(PDF)

S6 Table Linear mixed model regression systolic blood pressure. (PDF)

S7 Table Linear mixed model regression on diastolic blood pressure. (PDF)

S8 Table Results of regression of offspring genotype for systolic blood pressure. A linear regression was performed of the offspring genotype at each time of measurement (birth, 1, 2, 3 and 5y) with sex and BMI as adjustment. The linear mixed-effect regression model was performed of the offspring genotype adjusted by sex and BMI as fixed effect and by the intercept and age as random effect.
(PDF)

S9 Table Results of regression of offspring genotype for diastolic blood pressure. A linear regression was performed of the offspring genotype at each time of measurement (birth, 1, 2, 3 and 5y) with sex and BMI as adjustment. The linear mixed-effect regression model was performed of the offspring genotype adjusted by sex and BMI as fixed effect and by the intercept and age as random effect.
(PDF)

S1 Fig Sample size evolution from birth to 5 years for the child only, the duos and trios. A) For the regression covariates only, B) For the SBP analysis and C) For the DBP analysis.
(PDF)

S2 Fig Child SBP and DBP evolution across the follow up. (PDF)

S3 Fig 80% statistical power curves in the FAMILY study. (PDF)

## References

[1] Lawes CM, Vander Hoorn S, Rodgers A, International Society of H (2008) Global burden of blood-pressure-related disease, 2001. Lancet 371: 1513–1518. doi: 10.1016/S0140-6736(08)60655-8 18456100 10.1016/S0140-6736(08)60655-8

[2] Danaei G, Finucane MM, Lin JK, Singh GM, Paciorek CJ, Cowan MJ, et al (2011) National, regional, and global trends in systolic blood pressure since 1980: systematic analysis of health examination surveys and epidemiological studies with 786 country-years and 5.4 million participants. Lancet 377: 568–577. doi: 10.1016/S0140-6736(10)62036-3 21295844 10.1016/S0140-6736(10)62036-3

[3] Kaplan NM, Opie LH (2006) Controversies in hypertension. Lancet 367: 168–176. doi: 10.1016/S0140-6736(06)67965-8 16413880 10.1016/S0140-6736(06)67965-8

[4] Slama M, Susic D, Frohlich ED (2002) Prevention of hypertension. Curr Opin Cardiol 17: 531–536. 12357131 10.1097/00001573-200209000-00014

[5] Ehret GB, Caulfield MJ (2013) Genes for blood pressure: an opportunity to understand hypertension. Eur Heart J 34: 951–961. doi: 10.1093/eurheartj/ehs455 23303660 10.1093/eurheartj/ehs455 PMC3612776

[6] Tragante V, Barnes MR, Ganesh SK, Lanktree MB, Guo W, Franceschini N, et al (2014) Gene-centric meta-analysis in 87,736 individuals of European ancestry identifies multiple blood-pressure-related loci. Am J Hum Genet 94: 349–360. doi: 10.1016/j.ajhg.2013.12.016 24560520 10.1016/j.ajhg.2013.12.016 PMC3951943

[7] Newton-Cheh C, Johnson T, Gateva V, Tobin MD, Bochud M, Coin L, et al (2009) Genome-wide association study identifies eight loci associated with blood pressure. Nat Genet 41: 666–676. doi: 10.1038/ng.361 19430483 10.1038/ng.361 PMC2891673

[8] Danese E, Montagnana M, Fava C (2013) Searching for genes involved in hypertension development in special populations: children and pre-eclamptic women. Where are we standing now? Clin Chem Lab Med 51: 2253–2269. doi: 10.1515/cclm-2013-0405 23969470 10.1515/cclm-2013-0405

[9] Parmar PG, Taal HR, Timpson NJ, Thiering E, Lehtimaki T, Marinelli M, et al (2016) International GWAS Consortium Identifies Novel Loci Associated with Blood Pressure in Children and Adolescents. Circ Cardiovasc Genet. 10.1161/CIRCGENETICS.115.001190 PMC5279885 26969751

[10] Oikonen M, Tikkanen E, Juhola J, Tuovinen T, Seppala I, Juonala M, et al (2011) Genetic variants and blood pressure in a population-based cohort: the Cardiovascular Risk in Young Finns study. Hypertension 58: 1079–1085. doi: 10.1161/HYPERTENSIONAHA.111.179291 22025373 10.1161/HYPERTENSIONAHA.111.179291 PMC3247907

[11] Howe LD, Parmar PG, Paternoster L, Warrington NM, Kemp JP, Briollais L, et al (2013) Genetic influences on trajectories of systolic blood pressure across childhood and adolescence. Circ Cardiovasc Genet 6: 608–614. doi: 10.1161/CIRCGENETICS.113.000197 24200906 10.1161/CIRCGENETICS.113.000197 PMC4759925

[12] Taal HR, Verwoert GC, Demirkan A, Janssens AC, Rice K, Ehret G, et al (2012) Genome-wide profiling of blood pressure in adults and children. Hypertension 59: 241–247. doi: 10.1161/HYPERTENSIONAHA.111.179481 22203742 10.1161/HYPERTENSIONAHA.111.179481 PMC3266432

[13] Whincup PH, Cook DG, Papacosta O (1992) Do maternal and intrauterine factors influence blood pressure in childhood? Arch Dis Child 67: 1423–1429. 1489219 10.1136/adc.67.12.1423 PMC1793969

[14] Mitsumata K, Saitoh S, Ohnishi H, Akasaka H, Miura T (2012) Effects of parental hypertension on longitudinal trends in blood pressure and plasma metabolic profile: mixed-effects model analysis. Hypertension 60: 1124–1130. doi: 10.1161/HYPERTENSIONAHA.112.201129 23006727 10.1161/HYPERTENSIONAHA.112.201129

[15] Knuiman MW, Divitini ML, Welborn TA, Bartholomew HC (1996) Familial correlations, cohabitation effects, and heritability for cardiovascular risk factors. Ann Epidemiol 6: 188–194. 8827153 10.1016/1047-2797(96)00004-x

[16] Mathias RA, Roy-Gagnon MH, Justice CM, Papanicolaou GJ, Fan YT, Pugh EW, et al (2003) Comparison of year-of-exam- and age-matched estimates of heritability in the Framingham Heart Study data. BMC Genet 4 Suppl 1: S36. 14975104 10.1186/1471-2156-4-S1-S36 PMC1866471

[17] Morrison KM, Atkinson SA, Yusuf S, Bourgeois J, McDonald S, McQueen MJ, et al (2009) The Family Atherosclerosis Monitoring In earLY life (FAMILY) study: rationale, design, and baseline data of a study examining the early determinants of atherosclerosis. Am Heart J 158: 533–539. doi: 10.1016/j.ahj.2009.07.005 19781411 10.1016/j.ahj.2009.07.005

[18] Voight BF, Kang HM, Ding J, Palmer CD, Sidore C, Chines PS, et al (2012) The metabochip, a custom genotyping array for genetic studies of metabolic, cardiovascular, and anthropometric traits. PLoS Genet 8: e1002793 doi: 10.1371/journal.pgen.1002793 22876189 10.1371/journal.pgen.1002793 PMC3410907

[19] Janssens AC, Moonesinghe R, Yang Q, Steyerberg EW, van Duijn CM, Khoury MJ (2007) The impact of genotype frequencies on the clinical validity of genomic profiling for predicting common chronic diseases. Genet Med 9: 528–535. doi: 10.1097GIM.0b013e31812eece0 17700391 10.1097/gim.0b013e31812eece0

[20] Dudbridge F (2013) Power and predictive accuracy of polygenic risk scores. PLoS Genet 9: e1003348 doi: 10.1371/journal.pgen.1003348 23555274 10.1371/journal.pgen.1003348 PMC3605113

[21] Li A, Meyre D (2013) Challenges in reproducibility of genetic association studies: lessons learned from the obesity field. Int J Obes (Lond) 37: 559–567. 22584455 10.1038/ijo.2012.82

[22] Sohani ZN, Anand SS, Robiou-du-Pont S, Morrison KM, McDonald SD, Atkinson SA, et al (2016) Risk Alleles in/near ADCY5, ADRA2A, CDKAL1, CDKN2A/B, GRB10, and TCF7L2 Elevate Plasma Glucose Levels at Birth and in Early Childhood: Results from the FAMILY Study. PLoS One 11: e0152107 doi: 10.1371/journal.pone.0152107 27049325 10.1371/journal.pone.0152107 PMC4822946

[23] Sitlani CM, Rice KM, Lumley T, McKnight B, Cupples LA, Avery CL, et al (2015) Generalized estimating equations for genome-wide association studies using longitudinal phenotype data. Stat Med 34: 118–130. doi: 10.1002/sim.6323 25297442 10.1002/sim.6323 PMC4321952

[24] Gibbons RD, Hedeker D, DuToit S (2010) Advances in analysis of longitudinal data. Annu Rev Clin Psychol 6: 79–107. doi: 10.1146/annurev.clinpsy.032408.153550 20192796 10.1146/annurev.clinpsy.032408.153550 PMC2971698

[25] Price AL, Patterson NJ, Plenge RM, Weinblatt ME, Shadick NA, Reich D (2006) Principal components analysis corrects for stratification in genome-wide association studies. Nat Genet 38: 904–909. doi: 10.1038/ng1847 16862161 10.1038/ng1847

[26] Bates D, Maechler M, Bolker B (2011) lme4: Linear mixed-effects models using S4 classes. R package version. 0.999375–42 ed.

[27] Purcell S, Neale B, Todd-Brown K, Thomas L, Ferreira MA, Bender D, et al (2007) PLINK: a tool set for whole-genome association and population-based linkage analyses. Am J Hum Genet 81: 559–575. doi: 10.1086/519795 17701901 10.1086/519795 PMC1950838

[28] Li A, Robiou-du-Pont S, Anand SS, Morrison KM, McDonald SD, Atkinson SA, et al (2016) Parental and child genetic contributions to obesity traits in early life based on 83 loci validated in adults: the FAMILY study. Pediatr Obes. 10.1111/ijpo.12205 28008729

[29] Ehret GB, Munroe PB, Rice KM, Bochud M, Johnson AD, Chasman DI, et al (2011) Genetic variants in novel pathways influence blood pressure and cardiovascular disease risk. Nature 478: 103–109. doi: 10.1038/nature10405 21909115 10.1038/nature10405 PMC3340926

[30] Wain LV, Verwoert GC, O'Reilly PF, Shi G, Johnson T, Johnson AD, et al (2011) Genome-wide association study identifies six new loci influencing pulse pressure and mean arterial pressure. Nat Genet 43: 1005–1011. doi: 10.1038/ng.922 21909110 10.1038/ng.922 PMC3445021

[31] Levy D, Ehret GB, Rice K, Verwoert GC, Launer LJ, Dehghan A, et al (2009) Genome-wide association study of blood pressure and hypertension. Nat Genet 41: 677–687. doi: 10.1038/ng.384 19430479 10.1038/ng.384 PMC2998712

[32] Lowry WE, Huang J, Ma YC, Ali S, Wang D, Williams DM, et al (2002) Csk, a critical link of g protein signals to actin cytoskeletal reorganization. Dev Cell 2: 733–744. 12062086 10.1016/s1534-5807(02)00175-2

[33] Flavahan S, Flavahan NA (2014) The atypical structure and function of newborn arterial endothelium is mediated by Rho/Rho kinase signaling. Am J Physiol Heart Circ Physiol 307: H628–632. doi: 10.1152/ajpheart.00327.2014 24951756 10.1152/ajpheart.00327.2014 PMC4137119

[34] Moreno-Dominguez A, El-Yazbi AF, Zhu HL, Colinas O, Zhong XZ, Walsh EJ, et al (2014) Cytoskeletal Reorganization Evoked by Rho-associated kinase- and Protein Kinase C-catalyzed Phosphorylation of Cofilin and Heat Shock Protein 27, Respectively, Contributes to Myogenic Constriction of Rat Cerebral Arteries. J Biol Chem 289: 20939–20952. doi: 10.1074/jbc.M114.553743 24914207 10.1074/jbc.M114.553743 PMC4110300

[35] Xi B, Zhao X, Chandak GR, Shen Y, Cheng H, Hou D, et al (2013) Influence of obesity on association between genetic variants identified by genome-wide association studies and hypertension risk in Chinese children. Am J Hypertens 26: 990–996. doi: 10.1093/ajh/hpt046 23591986 10.1093/ajh/hpt046

[36] Vinck WJ, Fagard RH, Loos R, Vlietinck R (2001) The impact of genetic and environmental influences on blood pressure variance across age-groups. J Hypertens 19: 1007–1013. 11403347 10.1097/00004872-200106000-00003

[37] Fowler SP, Puppala S, Arya R, Chittoor G, Farook VS, Schneider J, et al (2013) Genetic epidemiology of cardiometabolic risk factors and their clustering patterns in Mexican American children and adolescents: the SAFARI Study. Hum Genet 132: 1059–1071. doi: 10.1007/s00439-013-1315-2 23736306 10.1007/s00439-013-1315-2 PMC3845827

[38] Snieder H, Harshfield GA, Treiber FA (2003) Heritability of blood pressure and hemodynamics in African- and European-American youth. Hypertension 41: 1196–1201. doi: 10.1161/01.HYP.0000072269.19820.0D 12719445 10.1161/01.HYP.0000072269.19820.0D

[39] Simino J, Shi G, Bis JC, Chasman DI, Ehret GB, Gu X, et al (2014) Gene-age interactions in blood pressure regulation: a large-scale investigation with the CHARGE, Global BPgen, and ICBP Consortia. Am J Hum Genet 95: 24–38. doi: 10.1016/j.ajhg.2014.05.010 24954895 10.1016/j.ajhg.2014.05.010 PMC4085636

[40] Sovio U, Mook-Kanamori DO, Warrington NM, Lawrence R, Briollais L, Palmer CN, et al (2011) Association between common variation at the FTO locus and changes in body mass index from infancy to late childhood: the complex nature of genetic association through growth and development. PLoS Genet 7: e1001307 doi: 10.1371/journal.pgen.1001307 21379325 10.1371/journal.pgen.1001307 PMC3040655

[41] Leon DA, Koupil I, Mann V, Tuvemo T, Lindmark G, Mohsen R, et al (2005) Fetal, developmental, and parental influences on childhood systolic blood pressure in 600 sib pairs: the Uppsala Family study. Circulation 112: 3478–3485. doi: 10.1161/CIRCULATIONAHA.104.497610 16286592 10.1161/CIRCULATIONAHA.104.497610

