# Supplementary material for: Parental and offspring contribution of genetic markers of adult blood pressure in early life: The FAMILY study
Source: PLoS One. 2017 Oct 18;12(10):e0186218. doi: 10.1371/journal.pone.0186218 (PMC5646805; doi:10.1371/journal.pone.0186218)
Supplement: S1 Table — MAF, Minor Allele Frequency in the studied population. GWAS, Genome-Wide Association Study. SNP, Single Nucleotide Polymorphism. CHR, Chromosome. SBP, Systolic Blood Pressure. DBP, Diastolic Blood Pressure. (PDF) [file pone.0186218.s003.pdf]

Table S1: Characteristics of the 33 selected SNPs.

Gene Name	GWAS SNP	Proxy SNP	CHR	r^2	Proxy minor allele	Proxy major allele	MAF	Risk allele SBP	Risk allele DBP	Traits
MTHFR	rs17367504		1		G	A	0.161	A	A	DBP[1, 2], SBP[1-3]
MOV10	rs2932538		1		A	G	0.266	G	G	DBP[2], SBP[2]
PDE1A	rs16823124	rs1438065	2	1	A	G	0.303	NA	A	DBP[4]
SLC4A7	rs13082711		3		G	A	0.250	NA	G	DBP[1, 2]
MECOM	rs419076	rs223102	3	1	G	A	0.475	G	G	DBP[1, 2], SBP[1, 2]
ULK4	rs9815354	rs1717017	3	1	C	A	0.177	NA	C	DBP[2, 5, 6]
SLC39A8	rs13107325		4		A	G	0.071	G	G	DBP[1, 2], SBP[1, 2]
FGF5	rs1458038		4		A	G	0.291	A	A	DBP[1-3], SBP[1, 2]
NPR3	rs1173771		5		A	G	0.387	G	G	DBP[2], SBP[1, 2]
EBF1	rs11953630	rs12187017	5	1	A	G	0.354	G	G	DBP[2, 6], SBP[6]
HFE	rs1799945		6		G	C	0.154	G	G	DBP[1, 2, 7], SBP[1, 2]
BAG6	rs805303		6		A	G	0.379	G	G	DBP[2], SBP[2]
PIK3CG	rs17477177	rs12705390	7	1	A	G	0.194	A	NA	SBP[1]
CYP17A1	rs11191548		10		G	A	0.091	A	A	DBP[1, 2, 6], SBP[1-3, 5, 6]
C10orf107	rs4590817		10		C	G	0.151	G	G	DBP[2, 3], SBP[2]
PLCE1	rs932764		10		G	A	0.404	G	NA	SBP[1, 2]
SOX6	rs2014408	rs11023909	11	0.96	G	A	0.210	G	NA	SBP[7]
RELA	rs3741378		11		A	G	0.139	G	NA	SBP[4]
PLEKHA7	rs381815		11		A	G	0.296	A	A	DBP[1, 2], SBP[1, 2, 5]
ARGAP42	rs633185		11		G	C	0.283	C	C	DBP [1, 2], SBP [1, 2]
LSP1	rs661348		11		G	A	0.432	G	NA	SBP[7]
ADM	rs7129220		11		A	G	0.113	A	NA	SBP[2]
NUCB2	rs757081		11		G	C	0.339	G	NA	SBP[4]
TBX3	rs2384550		12		A	G	0.348	NA	G	DBP[5]
ATP2B1	rs2681472		12		G	A	0.171	A	A	DBP[1, 5], SBP[1, 5, 7]
SH2B3	rs3184504		12		G	A	0.495	A	A	DBP[1-3, 5], SBP[1, 2, 5]
CSK	rs1378942		15		C	A	0.336	C	C	DBP[1-3], SBP[1-3, 5]
FES	rs2521501		15		A	T	0.328	A	A	DBP[2], SBP[2]
ZNF652	rs12940887		17		A	G	0.359	A	A	DBP[2, 3], SBP[2]
PLCD3	rs12946454		17		T	A	0.267	T	NA	SBP[3]
GOSR2	rs17608766		17		G	A	0.121	G	NA	SBP[1, 2]
JAG1	rs1327235		20		G	A	0.473	G	G	DBP[1, 2], SBP[2]
ZNF831	rs6015450		20		G	A	0.119	G	G	DBP[1, 2], SBP[1, 2]

MAF, Minor Allele Frequency in the studied population. GWAS, Genome-Wide Association Study. SNP, Single Nucleotide Polymorphism. CHR, Chromosome. SBP, Systolic Blood Pressure. DBP, Diastolic Blood Pressure.

1. Wain LV, Verwoert GC, O'Reilly PF, Shi G, Johnson T, Johnson AD, et al. Genome-wide association study identifies six new loci influencing pulse pressure and mean arterial pressure. Nature genetics. 2011;43(10):1005-11. doi: 10.1038/ng.922. PubMed PMID: 21909110; PubMed Central PMCID: PMC3445021.

2. International Consortium for Blood Pressure Genome-Wide Association S, Ehret GB, Munroe PB, Rice KM, Bochud M, Johnson AD, et al. Genetic variants in novel pathways influence blood pressure and cardiovascular disease risk. Nature. 2011;478(7367):103-9. doi: 10.1038/nature10405. PubMed PMID: 21909115; PubMed Central PMCID: PMC3340926.

3. Newton-Cheh C, Johnson T, Gateva V, Tobin MD, Bochud M, Coin L, et al. Genome-wide association study identifies eight loci associated with blood pressure. Nature genetics. 2009;41(6):666-76. doi: 10.1038/ng.361. PubMed PMID: 19430483; PubMed Central PMCID: PMC2891673.

4. Tragante V, Barnes MR, Ganesh SK, Lanktree MB, Guo W, Franceschini N, et al. Gene-centric meta-analysis in 87,736 individuals of European ancestry identifies multiple blood-pressure-related loci. American journal of human genetics. 2014;94(3):349-60. doi: 10.1016/j.ajhg.2013.12.016. PubMed PMID: 24560520; PubMed Central PMCID: PMC3951943.

5. Levy D, Ehret GB, Rice K, Verwoert GC, Launer LJ, Dehghan A, et al. Genome-wide association study of blood pressure and hypertension. Nature genetics. 2009;41(6):677-87. doi: 10.1038/ng.384. PubMed PMID: 19430479; PubMed Central PMCID: PMC2998712.

6. Franceschini N, Fox E, Zhang Z, Edwards TL, Nalls MA, Sung YJ, et al. Genome-wide association analysis of blood-pressure traits in African-ancestry individuals reveals

common associated genes in African and non-African populations. American journal of human genetics. 2013;93(3):545-54. doi: 10.1016/j.ajhg.2013.07.010. PubMed PMID: 23972371; PubMed Central PMCID: PMC3769920.

7. Ganesh SK, Tragante V, Guo W, Guo Y, Lanktree MB, Smith EN, et al. Loci influencing blood pressure identified using a cardiovascular gene-centric array. Human molecular genetics. 2013;22(8):1663-78. doi: 10.1093/hmg/dds555. PubMed PMID: 23303523; PubMed Central PMCID: PMC3657476.
