# Supplementary material for: Parental and offspring contribution of genetic markers of adult blood pressure in early life: The FAMILY study
Source: PLoS One. 2017 Oct 18;12(10):e0186218. doi: 10.1371/journal.pone.0186218 (PMC5646805; doi:10.1371/journal.pone.0186218)
Supplement: S2 Table — SNP: Single Nucleotide Polymorphism. CHR: Chromosome. CR: Call Rate. HWE: P-value resulting of Hardy Weinberg Equilibrium test. (PDF) [file pone.0186218.s004.pdf]

Table S2: Genotype count, call rate and Hardy Weinberg Equilibrium (HEW) test for the FAMILY population.

Gene	SNP	Proxy	CHR	CR	Minor allele	Major allele	GENOTYPE	HWE
MTHFR	rs17367504		1	100.00	G	A	29/255/697	0.341
MOV10	rs2932538		1	100.00	A	G	78/358/545	8.28×10^{-2}
PDE1A	rs16823124	rs1438065	2	100.00	A	G	85/426/470	0.450
SLC4A7	rs13082711		3	100.00	G	A	48/383/550	8.32×10^{-2}
MECOM	rs419076	rs223102	3	100.00	G	A	37/275/669	0.191
ULK4	rs9815354	rs1717017	3	100.00	C	A	220/489/272	1.00
SLC39A8	rs13107325		4	100.00	A	G	86/400/495	0.699
FGF5	rs1458038		4	100.00	A	G	7/124/850	0.321
NPR3	rs1173771		5	100.00	A	G	144/474/363	0.638
EBF1	rs11953630	rs12187017	5	100.00	A	G	128/439/414	0.486
HFE	rs1799945		6	100.00	G	C	22/253/706	1.00
BAG6	rs805303		6	100.00	A	G	152/454/375	0.459
PIK3CG	rs17477177	rs12705390	7	100.00	A	G	39/306/636	0.761
CYP17A1	rs11191548		10	100.00	G	A	26/244/711	0.382
C10orf107	rs4590817		10	99.15	C	G	164/452/353	0.350
PLCE1	rs932764		10	100.00	G	A	8/164/809	1.00
SOX6	rs2014408	rs11023909	11	100.00	G	A	179/488/314	0.696
RELA	rs3741378		11	100.00	A	G	16/187/778	0.257
PLEKHA7	rs381815		11	100.00	A	G	53/311/617	0.105
ARGAP42	rs633185		11	99.93	G	C	64/445/472	2.56×10^{-3}
LSP1	rs661348		11	99.93	G	A	109/441/431	0.830
ADM	rs7129220		11	100.00	A	G	18/233/730	1.00
NUCB2	rs757081		11	100.00	G	C	77/404/500	0.754
TBX3	rs2384550		12	100.00	A	G	26/286/669	0.575
ATP2B1	rs2681472		12	100.00	G	A	257/466/258	0.125
SH2B3	rs3184504		12	99.93	G	A	129/424/428	0.140
CSK	rs1378942		15	99.93	C	A	125/430/426	0.324
FES	rs2521501		15	99.93	A	T	93/452/436	0.127
ZNF652	rs12940887		17	99.93	A	G	73/370/537	0.410
PLCD3	rs12946454		17	100.00	T	A	15/209/757	0.881
GOSR2	rs17608766		17	100.00	G	A	116/465/400	0.296
JAG1	rs1327235		20	100.00	G	A	218/492/271	0.898
ZNF831	rs6015450		20	100.00	G	A	11/209/761	0.538

SNP: Single Nucleotide Polymorphism. CHR: Chromosome. CR: Call Rate. HWE: P-value resulting of Hardy Weinberg Equilibrium test.
