# Supplementary material for: Parental and offspring contribution of genetic markers of adult blood pressure in early life: The FAMILY study
Source: PLoS One. 2017 Oct 18;12(10):e0186218. doi: 10.1371/journal.pone.0186218 (PMC5646805; doi:10.1371/journal.pone.0186218)
Supplement: S3 Table — (PDF) [file pone.0186218.s005.pdf]

Table S3: Genotype count and Hardy Weinberg Equilibrium test (HWE) for mothers and fathers

SNP	Minor Allele	Major Allele	Mother			Father		
			MAF	GENO	HWE P-val	MAF	GENO	HWE P-val
rs17367504	G	A	0.1508	18/154/458	0.2743	0.1752	11/101/239	0.8554
rs2932538	A	G	0.2627	53/225/352	5.09×10^{-2}	0.2607	25/133/193	0.7817
rs1438065	A	G	0.296	52/269/309	0.5676	0.3177	33/157/161	0.6227
rs13082711	G	A	0.2556	32/258/340	5.99×10^{-2}	0.2236	16/125/210	0.7585
rs1717017	C	A	0.1817	23/183/424	0.5914	0.1709	14/92/245	0.1846
rs223102	G	A	0.4762	140/320/170	0.6898	0.4687	80/169/102	0.5221
rs1458038	A	G	0.2881	51/261/318	0.8464	0.2977	35/139/177	0.3097
rs13107325	A	G	0.07302	4/84/542	0.5652	0.06553	3/40/308	0.1755
rs1173771	A	G	0.3841	89/306/235	0.5556	0.396	55/168/128	1
rs12187017	A	G	0.3683	80/304/246	0.3919	0.3291	48/135/168	1.59×10^{-2}
chr6_26199158	G	C	0.1516	16/159/455	0.6417	0.151	6/94/251	0.5327
rs805303	A	G	0.3698	90/286/254	0.5495	0.416	62/168/121	0.826
rs12705390	A	G	0.1929	25/193/412	0.7009	0.2009	14/113/224	1
chr10:63137559	C	G	0.1556	20/156/454	0.1703	0.1425	6/88/257	0.8267
chr10:95885930	G	A	0.4034	111/279/231	9.60×10^{-2}	0.4009	53/173/122	0.5771
rs11191548	G	A	0.09683	6/110/514	1	0.08262	2/54/295	1
rs661348	G	A	0.4341	113/321/196	0.3732	0.4259	66/167/118	0.6622
rs7129220	A	G	0.1119	9/123/498	0.6867	0.1111	7/64/280	0.1695
rs11023909	G	A	0.2087	32/199/399	0.2777	0.2194	21/112/218	0.212
rs381815	A	G	0.2944	41/289/300	9.61×10^{-3}	0.2877	23/156/172	0.1512
rs757081	G	C	0.3389	71/285/274	0.859	0.3305	38/156/157	1
rs3741378	A	G	0.1365	13/146/471	0.6146	0.1382	5/87/259	0.6523
chr11:100098748	G	C	0.2873	49/264/317	0.6267	0.2792	28/140/183	0.8945
rs2681472	G	A	0.173	18/182/430	0.8898	0.1709	8/104/239	0.457
rs3184504	A	G	0.4992	161/307/162	0.5244	0.5	96/159/96	8.74×10^{-2}
rs2384550	A	G	0.3421	83/265/282	0.1108	0.3575	46/159/146	0.8164
rs1378942	C	A	0.3484	80/279/271	0.5395	0.3433	45/151/155	0.4075
chr15:89238392	A	T	0.3381	64/298/268	0.1815	0.302	29/154/168	0.5266
chr17:40563647	T	A	0.2719	42/258/329	0.4204	0.2479	31/112/208	9.45×10^{-3}
rs17608766	G	A	0.1286	12/138/480	0.5923	0.1097	3/71/277	0.7829
rs12940887	A	G	0.3532	71/303/256	0.2219	0.359	45/162/144	1
rs1327235	G	A	0.4786	144/315/171	1	0.463	74/177/100	0.8305
rs6015450	G	A	0.127	10/140/480	1	0.1011	1/69/281	0.2315
