# Supplementary material for: Parental and offspring contribution of genetic markers of adult blood pressure in early life: The FAMILY study
Source: PLoS One. 2017 Oct 18;12(10):e0186218. doi: 10.1371/journal.pone.0186218 (PMC5646805; doi:10.1371/journal.pone.0186218)
Supplement: S4 Table — A linear regression was performed of the offspring genotype at each time of measurement (birth, 1, 2, 3 and 5y) with sex and BMI as adjustment. The linear mixed-effect regression model was performed of the offspring genotype adjusted by sex and BMI as fixed effect and by the intercept and age as random effect. (PDF) [file pone.0186218.s006.pdf]

Table S4: Results of regression of offspring genotype for Systolic Blood Pressure

			Mixed Model		
GENE	SNP	Risk allele	BETA	SE	P-value
MTHFR	rs17367504	A	-0.927	0.627	0.139
MOV10	rs2932538	G	0.165	0.524	0.753
MECOM	rs223102	G	-0.454	0.442	0.304
SLC39A8	rs13107325	G	-0.717	0.829	0.387
FGF5	rs1458038	A	-0.291	0.499	0.560
NPR3	rs1173771	G	0.436	0.450	0.332
EBF1	rs12187017	G	0.444	0.456	0.329
HFE	rs1799945	G	-0.273	0.603	0.650
BAG6	rs805303	G	-0.132	0.452	0.770
PIK3CG	rs12705390	A	-0.593	0.583	0.309
CYP17A1	rs11191548	A	0.407	0.775	0.600
C10orf107	rs4590817	G	-0.224	0.632	0.723
PLCE1	rs932764	G	0.639	0.457	0.162
SOX6	rs11023909	G	-1.035	0.561	6.50×10^{-2}
RELA	rs3741378	G	0.100	0.665	0.881
PLEKHA7	rs381815	A	-0.351	0.484	0.469
ARGAP42	rs633185	C	-0.104	0.483	0.830
LSP1	rs661348	G	-0.125	0.453	0.782
ADM	rs7129220	A	-0.374	0.702	0.594
NUCB2	rs757081	G	0.406	0.495	0.413
ATP2B1	rs2681472	A	0.699	0.580	0.228
SH2B3	rs3184504	A	0.423	0.452	0.348
CSK	rs1378942	C	**0.979**	**0.462**	**3.42×10^{-2}**
FES	rs2521501	A	0.751	0.469	0.110
ZNF652	rs12940887	A	-0.206	0.444	0.643
PLCD3	rs12946454	T	**-1.067**	**0.501**	**3.34×10^{-2}**
GOSR2	rs17608766	G	-1.070	0.697	0.124
JAG1	rs1327235	G	0.410	0.446	0.358
ZNF831	rs6015450	G	1.106	0.704	0.116
	GS		0.022	0.099	0.826

A linear regression was performed of the offspring genotype at each time of measurement (birth, 1, 2, 3 and 5y) with sex and BMI as adjustment. The linear mixed-effect regression model was performed of the offspring genotype adjusted by sex and BMI as fixed effect and by the intercept and age as random effect.
