# Supplementary material for: Parental and offspring contribution of genetic markers of adult blood pressure in early life: The FAMILY study
Source: PLoS One. 2017 Oct 18;12(10):e0186218. doi: 10.1371/journal.pone.0186218 (PMC5646805; doi:10.1371/journal.pone.0186218)
Supplement: S5 Table — A linear regression was performed of the offspring genotype at each time of measurement (birth, 1, 2, 3 and 5y) with sex and BMI as adjustment. The linear mixed-effect regression model was performed of the offspring genotype adjusted by sex and BMI as fixed effect and by the intercept and age as random effect. (PDF) [file pone.0186218.s007.pdf]

Table S5: Results of regression of offspring genotype for Diastolic Blood Pressure

			Mixed Model		
GENE	SNP	Risk allele	BETA	SE	P-value
MTHFR	rs17367504	A	-0.604	0.495	0.222
MOV10	rs2932538	G	-0.228	0.411	0.579
PDE1A	rs1438065	A	-0.183	0.385	0.635
SLC4A7	rs13082711	G	0.100	0.401	0.803
MECOM	rs223102	G	-0.127	0.348	0.716
ULK4	rs1717017	C	-0.123	0.463	0.790
SLC39A8	rs13107325	G	-0.649	0.652	0.319
FGF5	rs1458038	A	0.084	0.392	0.831
NPR3	rs1173771	G	0.162	0.355	0.648
EBF1	rs12187017	G	0.574	0.359	0.110
HFE	rs1799945	G	0.093	0.477	0.846
BAG6	rs805303	G	-0.131	0.356	0.714
CYP17A1	rs11191548	A	**1.713**	**0.605**	**4.61×10^{-3}**
C10orf107	rs4590817	G	0.208	0.493	0.674
PLEKHA7	rs381815	A	-0.691	0.379	6.86×10^{-2}
ARGAP42	rs633185	C	-0.267	0.382	0.484
TBX3	rs2384550	G	0.023	0.357	0.949
ATP2B1	rs2681472	A	0.010	0.459	0.983
SH2B3	rs3184504	A	0.278	0.356	0.435
CSK	rs1378942	C	0.089	0.369	0.809
FES	rs2521501	A	0.202	0.370	0.586
ZNF652	rs12940887	A	0.300	0.350	0.391
JAG1	rs1327235	G	-0.185	0.352	0.598
ZNF831	rs6015450	G	0.754	0.554	0.173
	GS		0.032	0.082	0.694

A linear regression was performed of the offspring genotype at each time of measurement (birth, 1, 2, 3 and 5y) with sex and BMI as adjustment. The linear mixed-effect regression model was performed of the offspring genotype adjusted by sex and BMI as fixed effect and by the intercept and age as random effect.
