# Supplementary material for: Parental and offspring contribution of genetic markers of adult blood pressure in early life: The FAMILY study
Source: PLoS One. 2017 Oct 18;12(10):e0186218. doi: 10.1371/journal.pone.0186218 (PMC5646805; doi:10.1371/journal.pone.0186218)
Supplement: S6 Table — (PDF) [file pone.0186218.s008.pdf]

Table S6: Linear mixed model regression systolic blood pressure

			Maternal effect						Paternal effect					
			Adjustment child			Adjustment father			Adjustment child			Adjustment mother		
GENE	SNP	Risk allele	Beta	SD	P-val	Beta	SD	P-val	Beta	SD	P-val	Beta	SD	P-val
MTHFR	rs17367504	A	**-1.974**	**0.770**	**1.04×10^{-2}**	-1.465	0.884	9.75×10^{-2}	**2.418**	**0.930**	**9.28×10^{-3}**	1.250	0.788	0.113
MOV10	rs2932538	G	0.363	0.634	0.567	-0.188	0.731	0.797	0.518	0.826	0.531	0.385	0.723	0.594
MECOM	rs223102	G	0.633	0.580	0.275	-0.550	0.633	0.385	-0.165	0.726	0.820	-0.291	0.603	0.630
SLC39A8	rs13107325	G	0.610	1.187	0.607	0.090	1.267	0.943	1.014	1.503	0.500	0.792	1.130	0.483
FGF5	rs1458038	A	-0.959	0.623	0.124	-0.286	0.693	0.680	-0.224	0.782	0.775	-0.084	0.694	0.903
NPR3	rs1173771	G	0.799	0.600	0.183	0.159	0.664	0.811	-0.636	0.680	0.350	-0.841	0.606	0.165
EBF1	rs12187017	G	-0.843	0.599	0.159	-0.952	0.661	0.150	0.175	0.708	0.804	0.333	0.629	0.596
HFE	rs1799945	G	-0.840	0.754	0.266	**-2.347**	**0.822**	**4.28×10^{-3}**	-0.045	1.031	0.965	0.130	0.910	0.887
BAG6	rs805303	G	0.284	0.567	0.617	-0.001	0.608	0.998	-0.937	0.712	0.188	-0.801	0.632	0.205
PIK3CG	rs12705390	A	-0.044	0.710	0.951	-0.468	0.803	0.560	1.056	0.860	0.219	0.840	0.801	0.294
CYP17A1	rs11191548	A	1.213	1.030	0.239	1.302	1.153	0.259	2.781	1.485	6.11×10^{-2}	2.244	1.180	5.72×10^{-2}
C10orf107	rs4590817	G	0.357	0.802	0.656	-0.132	0.803	0.870	0.962	0.992	0.332	1.021	0.922	0.268
PLCE1	rs932764	G	-0.556	0.543	0.305	0.745	0.627	0.234	-0.857	0.706	0.225	0.261	0.651	0.688
SOX6	rs11023909	G	0.284	0.694	0.682	0.056	0.706	0.937	-0.323	0.783	0.680	-0.764	0.708	0.281
RELA	rs3741378	G	-0.748	0.808	0.355	0.197	0.900	0.827	-0.646	0.990	0.514	-0.853	0.923	0.356
PLEKHA7	rs381815	A	**1.314**	**0.637**	**3.90×10^{-2}**	1.062	0.698	0.128	-0.410	0.833	0.623	-0.344	0.739	0.642
ARGAP42	rs633185	C	-0.757	0.645	0.240	-0.568	0.696	0.414	0.356	0.842	0.672	0.013	0.730	0.986
LSP1	rs661348	G	-0.191	0.574	0.740	-0.082	0.615	0.894	-0.016	0.721	0.982	-0.244	0.649	0.707
ADM	rs7129220	A	0.360	0.852	0.672	-0.827	0.888	0.351	-1.582	1.129	0.161	**-2.055**	**0.949**	**3.03×10^{-2}**
NUCB2	rs757081	G	0.018	0.626	0.977	-0.492	0.699	0.481	0.855	0.705	0.225	0.188	0.658	0.775
ATP2B1	rs2681472	A	0.175	0.766	0.820	0.949	0.841	0.259	-0.116	0.931	0.901	0.215	0.888	0.809
SH2B3	rs3184504	A	-0.429	0.563	0.446	0.803	0.595	0.177	-0.510	0.705	0.469	-0.127	0.613	0.836
CSK	rs1378942	C	-0.420	0.585	0.472	0.643	0.659	0.329	-0.371	0.779	0.634	0.275	0.656	0.675
FES	rs2521501	A	-0.489	0.585	0.403	0.019	0.644	0.976	0.769	0.750	0.305	0.864	0.696	0.214
ZNF652	rs12940887	A	0.475	0.624	0.446	0.886	0.688	0.198	-1.426	0.759	6.02×10^{-2}	-1.026	0.669	0.125
PLCD3	rs12946454	T	0.193	0.663	0.770	-0.384	0.706	0.586	0.539	0.802	0.501	0.453	0.712	0.525

			Maternal effect						Paternal effect					
			Adjustment child			Adjustment father			Adjustment child			Adjustment mother		
GENE	SNP	Risk allele	Beta	SD	P-val	Beta	SD	P-val	Beta	SD	P-val	Beta	SD	P-val
GOSR2	rs17608766	G	-0.407	0.858	0.635	0.119	1.032	0.908	-0.900	1.154	0.435	-0.527	1.019	0.605
JAG1	rs1327235	G	0.606	0.572	0.290	-0.021	0.655	0.975	-0.355	0.710	0.617	-0.305	0.617	0.621
ZNF831	rs6015450	G	0.429	0.868	0.622	0.199	0.957	0.835	-1.165	1.235	0.345	-0.399	1.136	0.725
	GS		-0.043	0.125	0.734	-0.034	0.136	0.804	-0.080	0.163	0.622	-0.045	0.143	0.750
