# Supplementary material for: Parental and offspring contribution of genetic markers of adult blood pressure in early life: The FAMILY study
Source: PLoS One. 2017 Oct 18;12(10):e0186218. doi: 10.1371/journal.pone.0186218 (PMC5646805; doi:10.1371/journal.pone.0186218)
Supplement: S7 Table — (PDF) [file pone.0186218.s009.pdf]

Table S7: Linear mixed model regression on diastolic blood pressure

			Maternal effect						Paternal effect					
			Adjustment child			Adjustment father			Adjustment child			Adjustment mother		
GENE	SNP	Risk allele	Beta	SD	P-val	Beta	SD	P-val	Beta	SD	P-val	Beta	SD	P-val
MTHFR	rs17367504	A	**-1.320**	**0.600**	**2.78×10^{-2}**	-1.382	0.758	6.82×10^{-2}	**1.888**	**0.796**	**1.77×10^{-2}**	0.494	0.678	0.466
MOV10	rs2932538	G	-0.017	0.496	0.973	-0.389	0.621	0.531	0.077	0.707	0.914	-0.280	0.623	0.653
PDE1A	rs1438065	A	0.468	0.493	0.342	0.193	0.629	0.759	-0.527	0.630	0.402	-0.425	0.580	0.464
SLC4A7	rs13082711	G	-0.446	0.518	0.389	-0.126	0.632	0.842	-0.316	0.694	0.648	-0.363	0.634	0.567
MECOM	rs223102	G	0.014	0.459	0.976	-0.678	0.541	0.210	0.326	0.622	0.601	0.174	0.513	0.735
ULK4	rs1717017	C	**1.384**	**0.562**	**1.39×10^{-2}**	0.943	0.688	0.171	-0.551	0.788	0.484	-1.120	0.711	0.115
SLC39A8	rs13107325	G	0.202	0.933	0.828	-0.197	1.092	0.857	-1.035	1.275	0.417	-0.247	0.963	0.797
FGF5	rs1458038	A	-0.856	0.489	8.01×10^{-2}	-0.527	0.592	0.374	0.120	0.666	0.857	0.483	0.591	0.414
NPR3	rs1173771	G	0.682	0.463	0.141	-0.154	0.561	0.783	0.301	0.582	0.605	-0.057	0.518	0.912
EBF1	rs12187017	G	**-1.013**	**0.466**	**2.98×10^{-2}**	-0.464	0.566	0.412	0.057	0.607	0.925	0.684	0.540	0.205
HFE	rs1799945	G	-0.651	0.586	0.267	**-1.656**	**0.700**	**1.80×10^{-2}**	0.251	0.879	0.775	0.492	0.779	0.527
BAG6	rs805303	G	0.547	0.444	0.217	0.678	0.522	0.194	-0.197	0.617	0.749	0.023	0.542	0.966
CYP17A1	rs11191548	A	-0.607	0.805	0.451	0.204	0.970	0.833	**3.602**	**1.241**	**3.70×10^{-3}**	**2.851**	**0.995**	**4.16×10^{-3}**
C10orf107	rs4590817	G	0.858	0.625	0.170	0.734	0.678	0.279	0.643	0.848	0.448	0.811	0.783	0.300
PLEKHA7	rs381815	A	0.876	0.495	7.64×10^{-2}	-0.066	0.597	0.912	-0.065	0.714	0.927	-0.773	0.633	0.222
ARGAP42	rs633185	C	-0.072	0.506	0.886	0.447	0.598	0.455	-0.367	0.718	0.609	-0.476	0.618	0.441
TBX3	rs2384550	G	-0.018	0.470	0.969	-0.472	0.550	0.391	0.524	0.654	0.423	0.087	0.572	0.879
ATP2B1	rs2681472	A	-0.869	0.598	0.146	-0.583	0.723	0.420	-0.292	0.798	0.715	-0.181	0.761	0.812
SH2B3	rs3184504	A	-0.214	0.440	0.627	0.157	0.512	0.759	-0.244	0.608	0.688	-0.087	0.523	0.868
CSK	rs1378942	C	-0.110	0.463	0.812	0.083	0.569	0.884	-0.173	0.671	0.797	0.113	0.564	0.842
FES	rs2521501	A	0.025	0.464	0.957	-0.066	0.549	0.905	1.005	0.646	0.120	0.874	0.593	0.140
ZNF652	rs12940887	A	-0.337	0.490	0.492	0.148	0.594	0.804	-0.222	0.654	0.734	0.034	0.575	0.953
JAG1	rs1327235	G	0.489	0.447	0.274	0.047	0.556	0.932	-0.550	0.602	0.361	-0.675	0.525	0.198
ZNF831	rs6015450	G	-0.342	0.680	0.615	0.007	0.809	0.993	-0.304	1.052	0.773	0.314	0.968	0.746
	GS		-0.017	0.102	0.868	-0.092	0.120	0.444	0.073	0.153	0.635	0.026	0.132	0.844
