# Supplementary material for: Parental and offspring contribution of genetic markers of adult blood pressure in early life: The FAMILY study
Source: PLoS One. 2017 Oct 18;12(10):e0186218. doi: 10.1371/journal.pone.0186218 (PMC5646805; doi:10.1371/journal.pone.0186218)
Supplement: S8 Table — A linear regression was performed of the offspring genotype at each time of measurement (birth, 1, 2, 3 and 5y) with sex and BMI as adjustment. The linear mixed-effect regression model was performed of the offspring genotype adjusted by sex and BMI as fixed effect and by the intercept and age as random effect. (PDF) [file pone.0186218.s010.pdf]

Table S8: Results of regression of offspring genotype for Systolic Blood Pressure

			Birth			1y			2y			3y			5y			Z-test P-value		
GENE	SNP	Risk allele	BETA	SE	P-val	BETA	SE	P-val	BETA	SE	P-val	BETA	SE	P-val	BETA	SE	P-val	P-val 0 to 1	P-val 1 to 2	P-val 2 to 3
MTHFR	rs17367504	A	0.151	1.500	0.920	-0.185	1.285	0.886	-1.871	1.147	0.104	-1.460	0.834	$8.1x10^{-2}$	-0.144	0.866	0.868	0.433	0.164	0.386
MOV10	rs2932538	G	0.812	1.263	0.521	1.345	1.027	0.191	-1.630	0.938	0.083	-0.066	0.691	0.924	0.548	0.721	0.447	0.371	**$1.6x10^{-2}$**	$9.0x10^{-2}$
MECOM	rs223102	G	0.238	1.095	0.828	-0.362	0.894	0.686	-0.874	0.787	0.268	-0.284	0.584	0.627	-0.564	0.619	0.363	0.336	0.334	0.274
SLC39A8	rs13107325	G	0.698	2.009	0.729	0.356	1.634	0.828	-1.236	1.471	0.401	-0.773	1.097	0.482	-1.099	1.146	0.338	0.447	0.235	0.401
FGF5	rs1458038	A	0.543	1.280	0.672	-0.306	0.969	0.753	-0.053	0.904	0.953	-0.398	0.657	0.545	-0.674	0.694	0.332	0.298	0.424	0.379
NPR3	rs1173771	G	0.493	1.224	0.688	0.513	0.869	0.555	0.829	0.801	0.301	0.176	0.594	0.768	-0.012	0.628	0.985	0.495	0.395	0.256
EBF1	rs12187017	G	-0.487	1.154	0.674	0.142	0.894	0.874	0.044	0.828	0.958	0.005	0.600	0.993	0.381	0.630	0.546	0.333	0.468	0.485
HFE	rs1799945	G	-0.385	1.534	0.802	**2.711**	**1.161**	**$2.0x10^{-2}$**	**-2.115**	**1.064**	**$4.8x10^{-2}$**	-0.324	0.808	0.688	-0.152	0.830	0.855	$5.4x10^{-2}$	**$1x10^{-3}$**	$9.0x10^{-2}$
BAG6	rs805303	G	-0.455	1.144	0.691	-1.167	0.887	0.189	-0.923	0.809	0.254	0.094	0.593	0.874	**1.303**	**0.621**	**$3.6x10^{-2}$**	0.311	0.420	0.155
PIK3CG	rs12705390	A	**3.427**	**1.397**	**$1.5x10^{-2}$**	-0.557	1.151	0.629	-1.163	1.028	0.258	-0.696	0.777	0.370	-1.085	0.807	0.180	**$1.4x10^{-2}$**	0.347	0.358
CYP17A1	rs11191548	A	**4.131**	**1.873**	**$2.9x10^{-2}$**	-0.482	1.577	0.760	0.051	1.429	0.972	-0.310	1.028	0.763	-0.448	1.055	0.672	**$3.0x10^{-2}$**	0.401	0.419
C10orf107	rs4590817	G	-2.637	1.863	0.159	1.653	1.248	0.186	-1.562	1.124	0.165	1.184	0.857	0.168	0.073	0.855	0.932	**$2.8x10^{-2}$**	**$2.8x10^{-2}$**	**$2.6x10^{-2}$**
PLCE1	rs932764	G	-1.112	1.188	0.350	1.103	0.923	0.233	**1.718**	**0.816**	**$3.6x10^{-2}$**	0.400	0.603	0.507	0.508	0.629	0.420	$7.0x10^{-2}$	0.309	$9.7x10^{-2}$
SOX6	rs11023909	G	-0.243	1.471	0.869	**-2.962**	**1.062**	**$6x10^{-3}$**	-0.874	1.011	0.388	-0.510	0.748	0.496	-0.110	0.775	0.887	$6.7x10^{-2}$	$7.7x10^{-2}$	0.386
RELA	rs3741378	G	-1.414	1.630	0.387	0.258	1.317	0.845	-0.426	1.172	0.716	-0.675	0.880	0.444	0.601	0.927	0.518	0.212	0.349	0.433
PLEKHA7	rs381815	A	0.263	1.246	0.833	-0.100	0.942	0.915	-0.148	0.891	0.868	-0.481	0.644	0.455	0.342	0.662	0.606	0.408	0.485	0.381
ARGAP42	rs633185	C	**-3.033**	**1.180**	**$1.1x10^{-2}$**	0.319	0.939	0.734	-0.107	0.864	0.901	-0.724	0.643	0.260	0.704	0.669	0.293	**$1.3x10^{-2}$**	0.369	0.283
LSP1	rs661348	G	-1.428	1.121	0.204	1.046	0.920	0.256	-1.247	0.805	0.122	-0.626	0.597	0.295	1.024	0.620	$9.9x10^{-2}$	**$4.4x10^{-2}$**	**$3.0x10^{-2}$**	0.268
ADM	rs7129220	A	0.451	1.925	0.815	-0.750	1.352	0.580	-0.302	1.229	0.806	-0.903	0.919	0.326	-0.272	0.985	0.783	0.305	0.403	0.348
NUCB2	rs757081	G	1.849	1.333	0.167	0.656	0.983	0.505	0.886	0.900	0.325	-0.290	0.648	0.654	-0.769	0.677	0.257	0.235	0.431	0.144
ATP2B1	rs2681472	A	**-2.824**	**1.290**	**$3.0x10^{-2}$**	0.996	1.077	0.356	0.073	1.050	0.944	0.271	0.781	0.728	0.300	0.808	0.710	**$1.2x10^{-2}$**	0.270	0.440
SH2B3	rs3184504	A	-1.680	1.246	0.179	0.760	0.865	0.380	**1.705**	**0.792**	**$3.2x10^{-2}$**	0.150	0.600	0.803	-0.057	0.624	0.927	$5.4x10^{-2}$	0.210	$5.9x10^{-2}$
CSK	rs1378942	C	-0.701	1.207	0.562	1.564	0.910	0.087	1.018	0.848	0.231	0.569	0.613	0.353	**1.285**	**0.631**	**$4.2x10^{-2}$**	$6.7x10^{-2}$	0.330	0.334
FES	rs2521501	A	**-3.187**	**1.207**	**$9x10^{-3}$**	0.508	0.906	0.576	0.994	0.838	0.236	0.119	0.631	0.850	**1.470**	**0.650**	**$2.4x10^{-2}$**	**$7x10^{-3}$**	0.347	0.202

			Birth			1y			2y			3y			5y			Z-test P-value		
GENE	SNP	Risk allele	BETA	SE	P-val	BETA	SE	P-val	BETA	SE	P-val	BETA	SE	P-val	BETA	SE	P-val	P-val 0 to 1	P-val 1 to 2	P-val 2 to 3
ZNF652	rs12940887	A	0.439	1.115	0.694	-0.801	0.863	0.354	-0.509	0.795	0.523	-0.131	0.587	0.824	0.103	0.622	0.868	0.190	0.402	0.351
PLCD3	rs12946454	T	-0.764	1.273	0.549	0.191	1.005	0.850	-0.693	0.903	0.443	-0.514	0.667	0.441	**-1.450**	**0.687**	**3.5x10^{-2}**	0.278	0.256	0.437
GOSR2	rs17608766	G	-1.505	1.772	0.397	2.036	1.383	0.142	-1.233	1.209	0.309	0.152	0.920	0.869	**-2.249**	**0.969**	**2.1x10^{-2}**	5.8x10^{-2}	**3.8x10^{-2}**	0.181
JAG1	rs1327235	G	0.970	1.179	0.412	-0.096	0.879	0.913	0.677	0.790	0.392	-0.411	0.589	0.486	-0.278	0.616	0.653	0.235	0.257	0.135
ZNF831	rs6015450	G	0.215	1.927	0.911	0.131	1.385	0.925	0.510	1.269	0.688	-0.518	0.920	0.573	1.791	0.966	6.4x10^{-2}	0.486	0.420	0.256
	GS		-0.397	0.263	0.134	0.290	0.197	0.142	-0.152	0.174	0.384	-0.195	0.129	0.130	0.140	0.140	0.320	**1.8x10^{-2}**	**4.7x10^{-2}**	0.420

A linear regression was performed of the offspring genotype at each time of measurement (birth, 1, 2, 3 and 5y) with sex and BMI as adjustment. The linear mixed-effect regression model was performed of the offspring genotype adjusted by sex and BMI as fixed effect and by the intercept and age as random effect.
