# Supplementary material for: Parental and offspring contribution of genetic markers of adult blood pressure in early life: The FAMILY study
Source: PLoS One. 2017 Oct 18;12(10):e0186218. doi: 10.1371/journal.pone.0186218 (PMC5646805; doi:10.1371/journal.pone.0186218)
Supplement: S9 Table — A linear regression was performed of the offspring genotype at each time of measurement (birth, 1, 2, 3 and 5y) with sex and BMI as adjustment. The linear mixed-effect regression model was performed of the offspring genotype adjusted by sex and BMI as fixed effect and by the intercept and age as random effect. (PDF) [file pone.0186218.s011.pdf]

Table S9: Results of regression of offspring genotype for Diastolic Blood Pressure

			Birth			1y			2y			3y			5y			Z-test P-value			
GENE	SNP	Risk allele	BETA	SE	P-val	BETA	SE	P-val	BETA	SE	P-val	BETA	SE	P-val	BETA	SE	P-val	P-val 0 to 1	P-val 1 to 2	P-val 2 to 3	P-v[illegible] 3 t[illegible]
MTHFR	rs17367504	A	-0.774	0.736	0.294	-0.164	0.862	0.849	-0.721	0.814	0.376	-0.327	0.575	0.570	-0.083	0.582	0.886	0.295	0.319	0.346	0.3[illegible]
MOV10	rs2932538	G	0.098	0.619	0.874	0.270	0.689	0.696	-0.912	0.665	0.171	-0.158	0.475	0.740	-0.333	0.484	0.492	0.427	0.109	0.178	0.3[illegible]
PDE1A	rs1438065	A	0.108	0.586	0.854	0.566	0.627	0.367	-0.329	0.622	0.597	-0.132	0.448	0.768	**0.939**	**0.450**	**3.8x10^{-2}**	0.297	0.155	0.399	**4.6x[illegible]**
SLC4A7	rs13082711	G	-0.425	0.623	0.496	-0.498	0.697	0.475	-0.675	0.633	0.287	0.240	0.456	0.599	0.129	0.468	0.783	0.469	0.425	0.120	0.4[illegible]
MECOM	rs223102	G	0.243	0.526	0.644	-0.218	0.598	0.716	**-1.313**	**0.554**	**1.8x10^{-2}**	-0.149	0.402	0.711	-0.342	0.416	0.412	0.281	9.0x10^{-2}	**4.4x10^{-2}**	0.3[illegible]
ULK4	rs1717017	C	-0.753	0.696	0.280	0.604	0.811	0.457	-0.804	0.727	0.269	0.138	0.538	0.797	0.144	0.545	0.792	0.102	9.8x10^{-2}	0.149	0.4[illegible]
SLC39A8	rs13107325	G	0.727	0.979	0.458	0.713	1.100	0.517	-1.255	1.040	0.228	-0.475	0.754	0.529	-1.344	0.768	8.1x10^{-2}	0.496	9.7x10^{-2}	0.272	0.2[illegible]
FGF5	rs1458038	A	0.352	0.586	0.548	0.664	0.647	0.306	-0.510	0.639	0.426	-0.130	0.452	0.775	-0.070	0.467	0.880	0.361	9.9x10^{-2}	0.314	0.4[illegible]
NPR3	rs1173771	G	0.853	0.558	0.127	-0.150	0.586	0.798	0.312	0.567	0.582	-0.047	0.408	0.908	-0.533	0.421	0.206	0.107	0.285	0.304	0.2[illegible]
EBF1	rs12187017	G	-0.348	0.545	0.523	-0.379	0.600	0.528	1.010	0.584	8.4x10^{-2}	0.422	0.412	0.306	-0.091	0.423	0.830	0.485	**4.8x10^{-2}**	0.205	0.1[illegible]
HFE	rs1799945	G	0.365	0.743	0.624	0.944	0.784	0.229	-1.063	0.755	0.160	0.081	0.556	0.885	0.011	0.558	0.985	0.296	**3.3x10^{-2}**	0.111	0.4[illegible]
BAG6	rs805303	G	-0.161	0.541	0.766	-0.436	0.596	0.464	-0.585	0.572	0.308	0.549	0.407	0.178	**1.005**	**0.416**	**1.6x10^{-2}**	0.366	0.429	5.3x10^{-2}	0.2[illegible]
CYP17A1	rs11191548	A	**2.694**	**0.884**	**2x10^{-3}**	0.234	1.060	0.825	0.516	1.011	0.610	0.170	0.707	0.810	0.315	0.708	0.657	**3.7x10^{-2}**	0.424	0.390	0.4[illegible]
C10orf107	rs4590817	G	0.567	0.739	0.443	0.825	0.839	0.326	-0.315	0.797	0.693	0.635	0.589	0.282	0.224	0.574	0.697	0.409	0.162	0.169	0.3[illegible]
PLEKHA7	rs381815	A	-1.029	0.580	7.7x10^{-2}	-0.759	0.629	0.229	-0.072	0.630	0.910	-0.403	0.442	0.362	-0.586	0.444	0.187	0.376	0.220	0.333	0.3[illegible]
ARGAP42	rs633185	C	-0.437	0.589	0.459	0.602	0.629	0.339	-0.641	0.611	0.294	-0.662	0.441	0.134	0.551	0.449	0.221	0.114	7.8x10^{-2}	0.489	**2.7x[illegible]**
TBX3	rs2384550	G	0.905	0.546	9.8x10^{-2}	-0.041	0.597	0.946	-0.427	0.567	0.451	0.440	0.407	0.281	-0.071	0.421	0.866	0.121	0.319	0.107	0.1[illegible]
ATP2B1	rs2681472	A	0.029	0.701	0.967	0.482	0.722	0.505	-1.034	0.741	0.164	-0.173	0.537	0.747	-0.130	0.543	0.810	0.326	7.1x10^{-2}	0.173	0.4[illegible]
SH2B3	rs3184504	A	0.055	0.548	0.920	-0.141	0.585	0.809	**1.205**	**0.561**	**3.2x10^{-2}**	-0.309	0.412	0.453	-0.434	0.418	0.301	0.403	**4.8x10^{-2}**	**1.5x10^{-2}**	0.4[illegible]
CSK	rs1378942	C	-0.243	0.565	0.667	0.983	0.612	0.109	0.316	0.601	0.599	-0.200	0.422	0.635	0.528	0.425	0.215	7.0x10^{-2}	0.218	0.241	0.1[illegible]
FES	rs2521501	A	-0.776	0.570	0.175	0.296	0.607	0.626	0.690	0.593	0.245	-0.265	0.434	0.542	0.710	0.438	0.106	9.9x10^{-2}	0.321	9.7x10^{-2}	5.7x[illegible]
ZNF652	rs12940887	A	-0.271	0.538	0.615	-0.104	0.578	0.857	-0.439	0.563	0.436	**0.981**	**0.400**	**1.5x10^{-2}**	0.543	0.417	0.193	0.416	0.339	**2.0x10^{-2}**	0.2[illegible]
JAG1	rs1327235	G	-0.115	0.540	0.832	-0.475	0.588	0.420	0.096	0.559	0.864	-0.606	0.404	0.134	-0.518	0.413	0.211	0.326	0.241	0.155	0.4[illegible]
ZNF831	rs6015450	G	-1.473	0.859	8.7x10^{-2}	0.074	0.927	0.936	0.618	0.897	0.491	-0.413	0.632	0.514	1.087	0.649	9.5x10^{-2}	0.110	0.337	0.174	**4.9x[illegible]**
	GS		-0.036	0.126	0.778	0.101	0.139	0.466	-0.215	0.132	0.103	-0.014	0.093	0.881	0.080	0.096	0.406	0.233	**4.9x10^{-2}**	0.106	0.2[illegible]

A linear regression was performed of the offspring genotype at each time of measurement (birth, 1, 2, 3 and 5y) with sex and BMI as adjustment. The linear mixed-effect regression model was performed of the offspring genotype adjusted by sex and BMI as fixed effect and by the intercept and age as random effect.
