# Supplementary material for: Parental and offspring contribution of genetic markers of adult blood pressure in early life: The FAMILY study
Source: PLoS One. 2017 Oct 18;12(10):e0186218. doi: 10.1371/journal.pone.0186218 (PMC5646805; doi:10.1371/journal.pone.0186218)

Figure S1. Sample size evolution from birth to 5 years for the child only, the duos and trios. A) For the regression covariates only, B) For the SBP analysis and C) For the DBP analysis.

A)

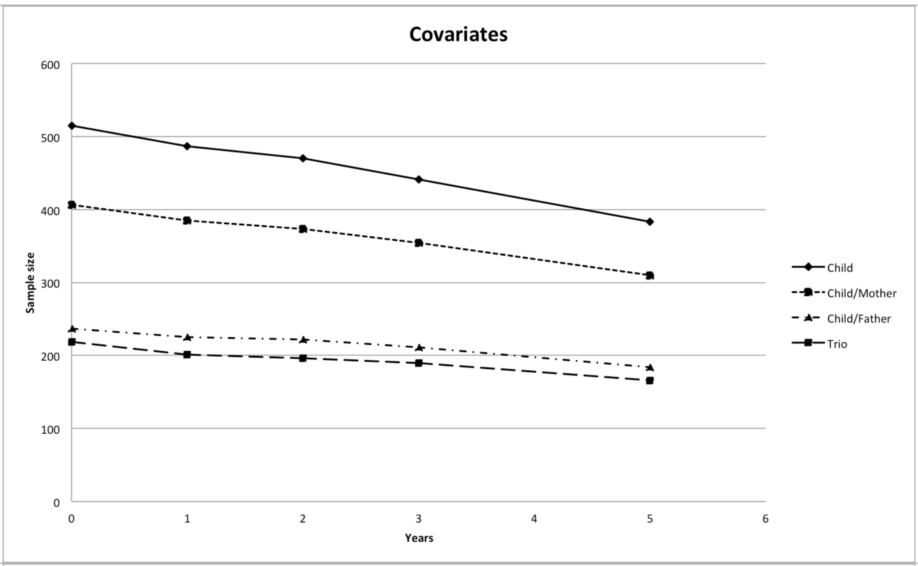

B)

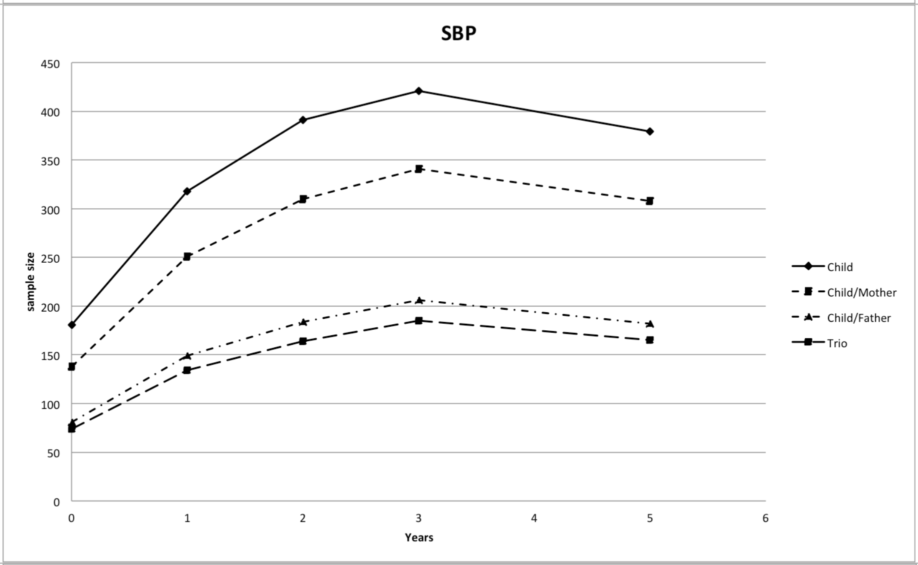

C)

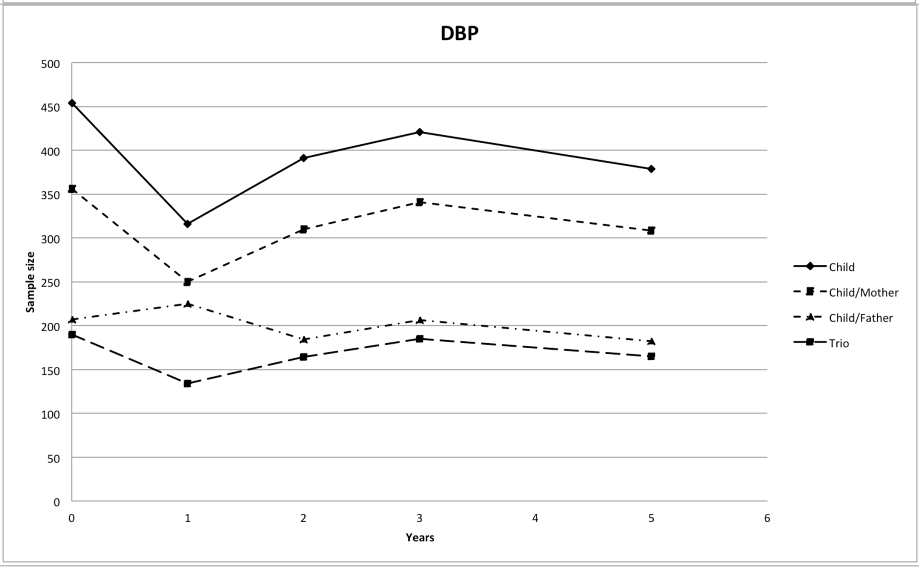

Supplement: S1 Fig — A) For the regression covariates only, B) For the SBP analysis and C) For the DBP analysis. (PDF) [file pone.0186218.s012.pdf]
