# Supplementary figures and images for: Parental and offspring contribution of genetic markers of adult blood pressure in early life: The FAMILY study
Source: PLoS One. 2017 Oct 18;12(10):e0186218. doi: 10.1371/journal.pone.0186218 (PMC5646805; doi:10.1371/journal.pone.0186218)

**Figure S2.** Child SBP and DBP evolution across the follow up

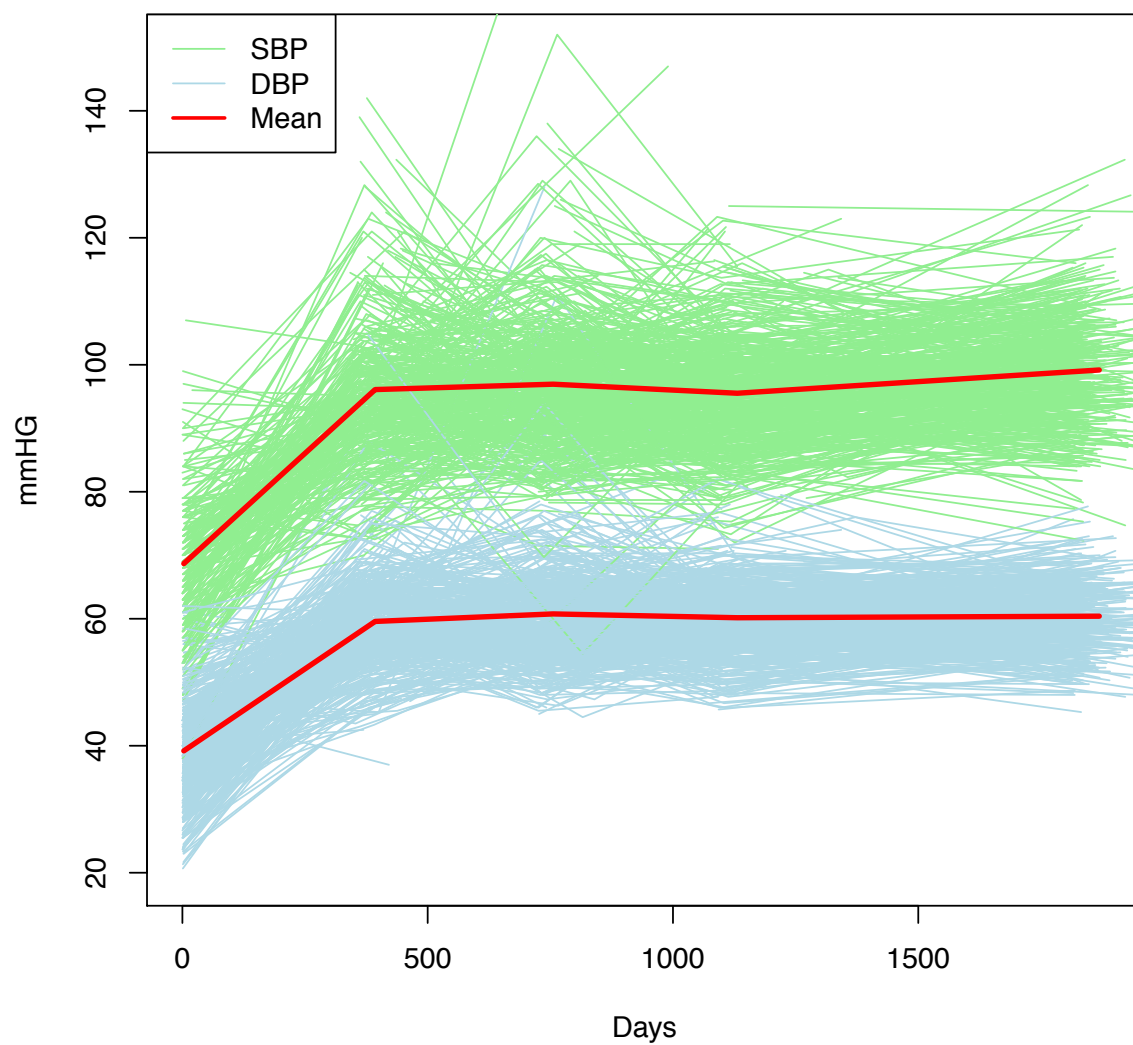

Supplement: S2 Fig — (PDF) [file pone.0186218.s013.pdf]
