# Supplementary material for: Parental and offspring contribution of genetic markers of adult blood pressure in early life: The FAMILY study
Source: PLoS One. 2017 Oct 18;12(10):e0186218. doi: 10.1371/journal.pone.0186218 (PMC5646805; doi:10.1371/journal.pone.0186218)
Supplement: S3 Fig — (PDF) [file pone.0186218.s014.pdf]

Figure S3. Curves highlighted the power of our study to reach 80% of power.

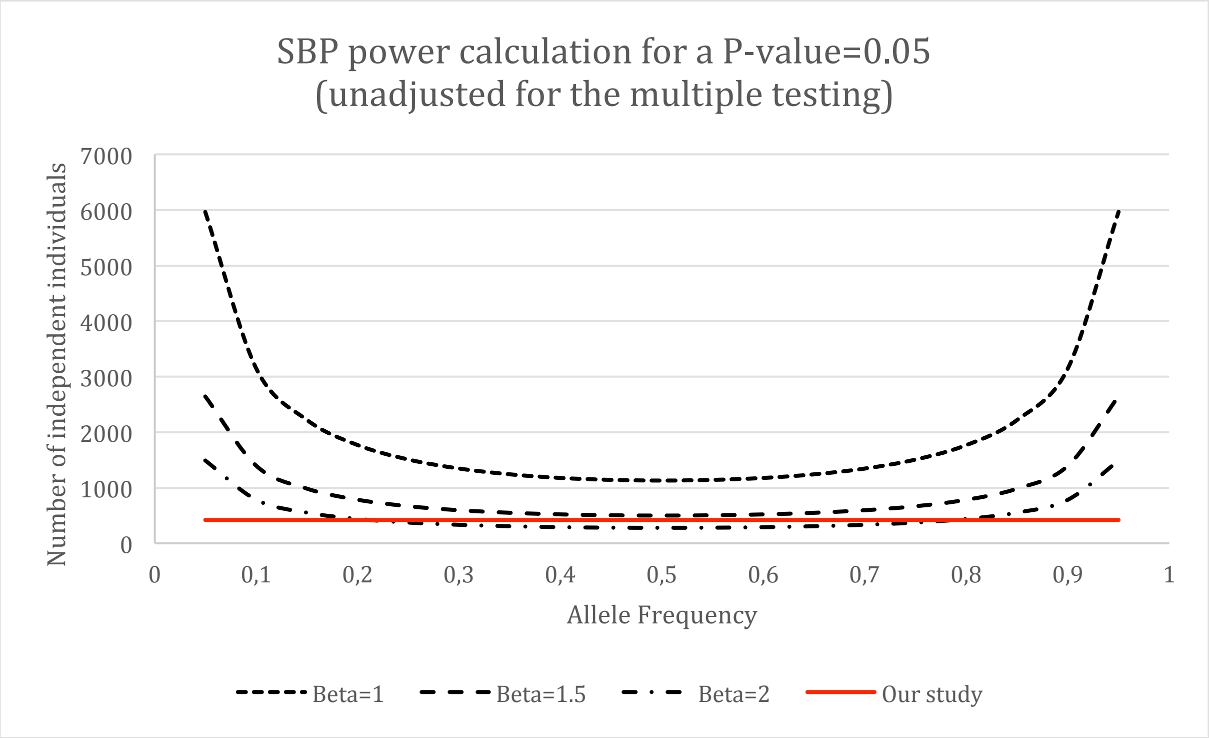

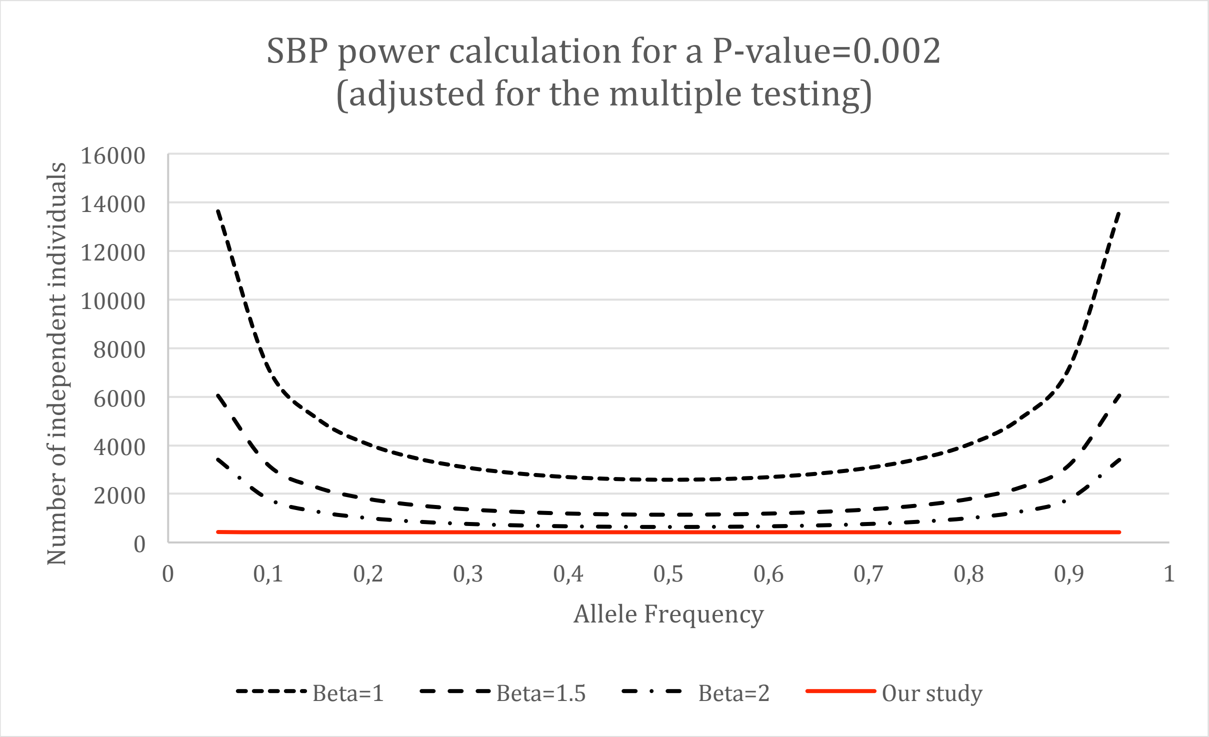

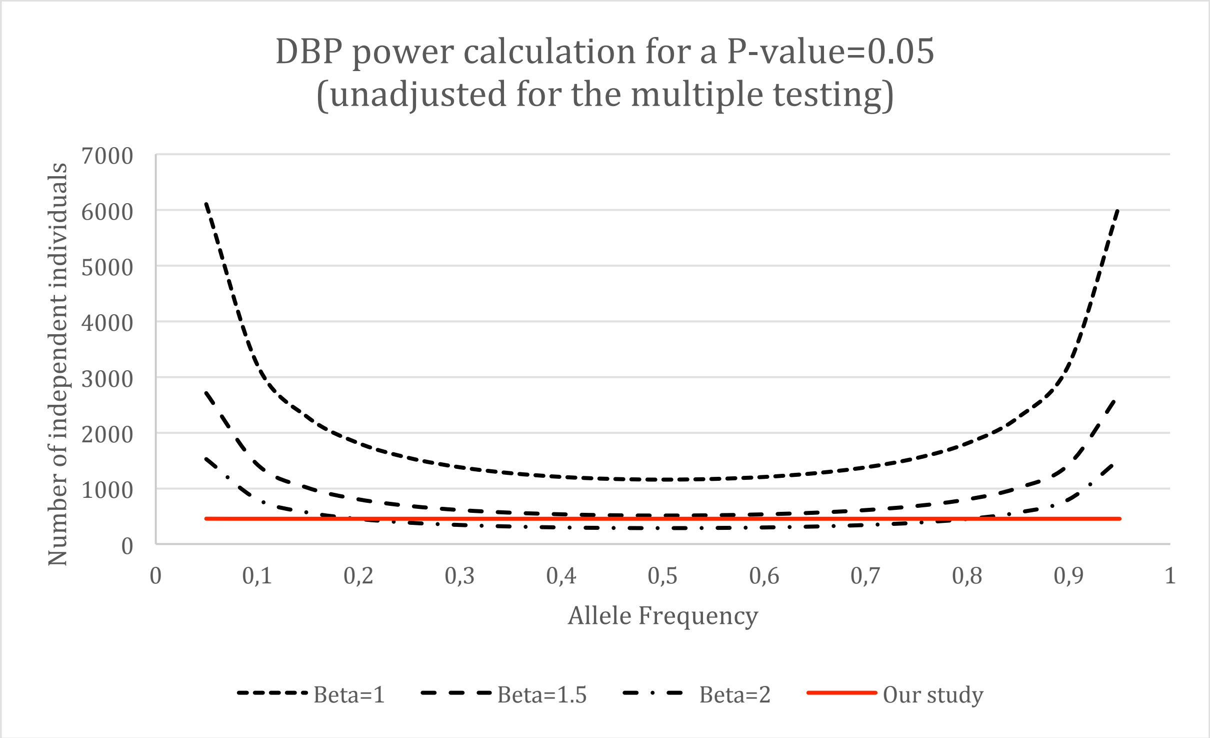
DBP power calculation for a P-value=0.05
(unadjusted for the multiple testing)
Number of independent individuals
7000
6000
5000
4000
3000
2000
1000
0
0
0,1
0,2
0,3
0,4
0,5
0,6
0,7
0,8
0,9
1
Allele Frequency
Beta=1
Beta=1.5
Beta=2
Our study

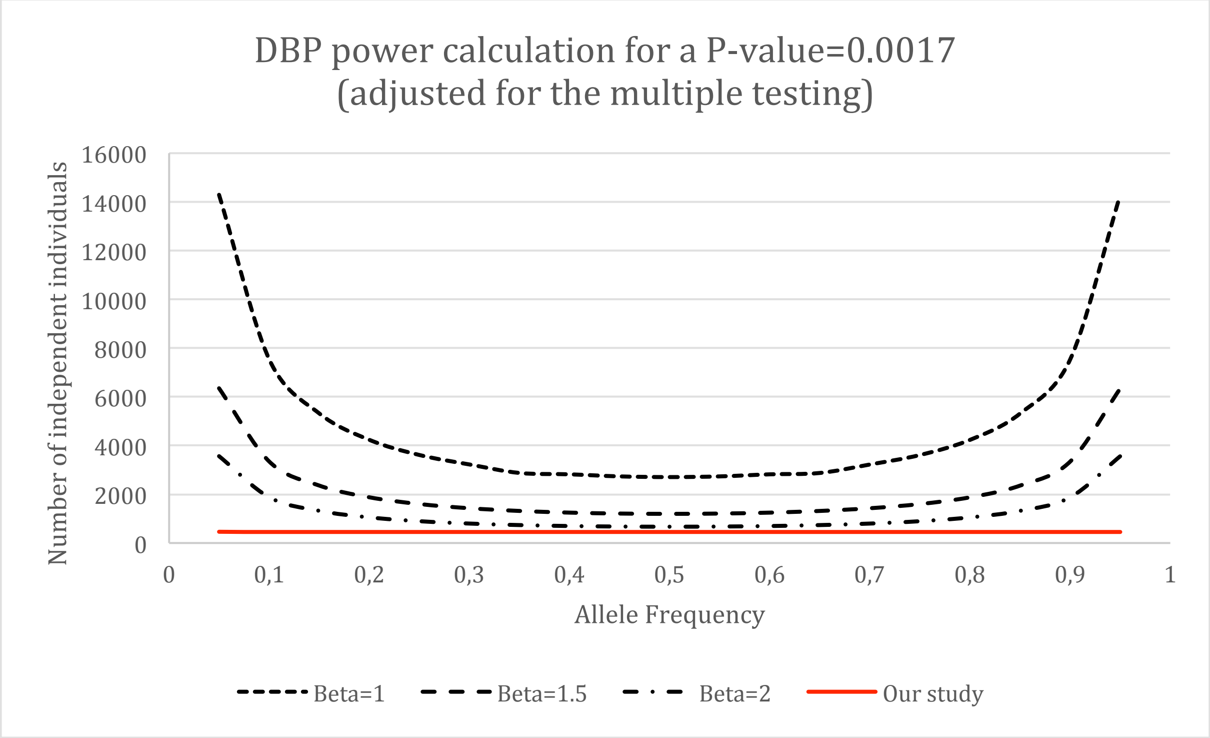
DBP power calculation for a P-value=0.0017
(adjusted for the multiple testing)
Number of independent individuals
16000
14000
12000
10000
8000
6000
4000
2000
0
0
0,1
0,2
0,3
0,4
0,5
0,6
0,7
0,8
0,9
1
Allele Frequency
Beta=1
Beta=1.5
Beta=2
Our study
